# Disentangling Response Magnitude and Relative MOS Sensor Fingerprints for Electronic-Nose Screening of Off-Odor in Table Olives

**DOI:** 10.3390/s26206605

**Published:** 2026-10-18

**Authors:** Ramiro Sánchez

**Affiliations:** Centro de Investigaciones Científicas y Tecnológicas de Extremadura (CICYTEX), Junta de Extremadura, Avda. Adolfo Suárez s/n, 06007 Badajoz, Spain; ramiro.sanchez@juntaex.es

**Keywords:** electronic nose, MOS sensors, signal representation, log-conductance ratio, centered log-ratio, response magnitude, table olives, off-odor, industrial screening, LDA

## Abstract

A multichannel metal-oxide semiconductor (MOS) electronic-nose response contains complementary information on overall response magnitude and the relative pattern among sensors. The most appropriate representation therefore depends on whether the aim is to distinguish volatile profiles, response levels, or both. This study compared three mathematically linked representations of the same six-channel NASYS S3+ response: global magnitude (M), centered log-ratio profiles (CLR), and per-sensor log-conductance ratios (LGR), which retain both components. Reference solutions varied both compound identity and concentration (four compounds, three levels, six sessions). The same framework was applied to 24 commercial cans of Californian-style black table olives preassigned by the supplier as conforming (C), mild (DS), or strong (DF) barnyard-like off-odor: 15 for calibration, six reserved before fitting, and three coded unknowns. Using the same linear discriminant analysis (LDA), CLR retained reference-compound class information better than M (balanced accuracy, BA, 0.83–0.89 vs. ≤0.56). In olives, grouped cross-validation favored M (BA 0.900), whereas LGR classified all six reserve cans and M only four; both results are descriptive within-campaign comparisons on 15 and 6 cans, not external validation. The three coded unknowns matched the concealed supplier labels. Signal representation should therefore be selected according to the analytical question rather than solely by calibration cross-validation performance.

## 1. Introduction

Table olives are produced and traded on a global scale, and sensory consistency is a central component of their commercial quality. The International Olive Council estimated world production at approximately 3.32 million tonnes for the 2024/25 crop year and forecast approximately 2.99 million tonnes for 2025/26 [1]. At this scale, routine quality control must distinguish normal product variability from changes that warrant sensory or analytical investigation. Instrumental screening cannot replace a trained sensory panel, but it can help prioritize lots, standardize preliminary decisions, and reduce the number of samples requiring more demanding analyses.

Off-odors may arise during fermentation, processing, storage, or packaging. Sensory analysis describes what is perceived, gas chromatography characterizes the volatile fraction, and an electronic nose records a multichannel response to the sample headspace. These approaches therefore address different but complementary aspects of product quality. Electronic noses have already been used to discriminate table olive quality [2], abnormal fermentations [3], sensory categories [4], and processing conditions in Californian-style black olives [5]. Beyond application-specific classification, recent work has also emphasized that signal preprocessing and validation design can influence model parsimony and generalization [6]. The S3+ platform itself has been used by independent groups for the longitudinal monitoring of fermented food processes [7].

Most classification studies ask whether sample groups can be separated. A less explicit question is why they separate. Two groups may differ because all sensors respond more strongly to one group while their relative response pattern remains nearly unchanged. Conversely, two groups may show similar overall response magnitude but differ in the relative balance among sensor channels. These situations are not equivalent: the first is dominated by global response magnitude, whereas the second is dominated by the relative sensor fingerprint. A third situation is also possible, in which both sources of information contribute to discrimination. The most appropriate signal representation should therefore depend on the analytical question—whether the aim is to distinguish differences primarily associated with response level, relative volatile-response pattern, or both—rather than simply on which representation produces the highest calibration performance.

This distinction is particularly relevant for cross-sensitive metal-oxide semiconductor (MOS) sensor arrays. Surface reactions with volatile compounds modify electrical conductance [8], but each sensor responds to multiple compounds and the same compound can affect several channels. The measured response therefore reflects the combined effects of volatile composition, overall stimulus strength, humidity, instrument state, and acquisition conditions. A MOS fingerprint is not a chromatogram and should not be interpreted as molecular identification. Nevertheless, the relative balance among channels can contain information associated with differences in the stimulus reaching the array, whereas their common response level contributes information about overall signal magnitude. Feature representation is consequently more than a technical preprocessing step: it determines which components of the physical sensor response are made available to the classifier. New sensing materials, such as carbon nanotube–titanium dioxide hybrid nanostructures, can improve the detection and discrimination of volatile organic compounds [9]; however, the question of how to represent multichannel responses applies to cross-sensitive sensor arrays regardless of their sensing materials.

Transfer across sessions and physical samples adds a second difficulty. MOS systems are sensitive to temporal variation and sensor drift. Long-term drift and compensation strategies have been studied extensively with gas-sensor arrays [10,11,12,13], while Palacín et al. showed that signal representation and classification performance can change over time even in a controlled volatile experiment [14]. Reference compounds can therefore provide a useful bridge between sensor behavior and food classification because both compound identity and stimulus level can be varied under controlled conditions. Teixeira et al., for example, combined defined aromatic substances with electronic-nose discrimination of olive oils differing in perceived fruitiness [15]. Controls are equally important: a water blank probes the measurement procedure and humidity-related response, whereas a fixed chemical mixture can reveal whether a nominally constant stimulus produces a stable multichannel response over time [16].

The present work addresses a simple but practically important question: which part of the MOS response should be preserved for a given classification problem? Signal preprocessing, baseline manipulation, feature extraction, array normalization, and pattern analysis are established components of electronic-nose methodology [17,18,19]. Here, three mathematically linked representations of the same six-channel response were compared. Global magnitude (M) compresses the array response into a single value and describes how strongly the array responds overall. The centered log-ratio profile (CLR), inspired by the centered log-ratio transformation used in compositional data analysis [20], removes the common log-scale magnitude term and retains the relative distribution of the response among sensors; the sensor responses are not interpreted as chemical proportions. Per-sensor log-conductance ratios (LGR) retain both global magnitude and relative sensor pattern. Thus, the algebraic decomposition itself is straightforward. The methodological contribution lies in comparing these representations experimentally under the same classifier and the same validation partitions, so that differences in performance can be related to the information retained rather than to changes in the modeling procedure.

Two connected experiments were designed around this question. First, four chemically distinct reference compounds were measured at three nominal concentration levels across six sessions. This design intentionally varied both compound class and stimulus level, allowing the behavior of M, CLR, and LGR to be compared when the analytical question concerns relative profile, response magnitude, or both. The same framework was then transferred to sealed commercial cans of Californian-style black table olives supplied in three categories: conforming, mild barnyard-like off-odor, and strong barnyard-like off-odor. In this real-food application, the target distinction was not among chemically defined reference compounds but between conforming samples and samples differing in supplier-assigned off-odor status and intensity. The applied objective was therefore binary screening of conforming versus off-odor categories, while the methodological objective was to determine whether the representation favored during calibration was also the one that transferred best to physical cans reserved before model fitting. The complete analysis was implemented in NECA ML, an in-house environment developed by the author at CICYTEX to integrate physical-sample grouping, signal representation, validation strategy, and prediction reporting within a single workflow. The novelty of this study lies in the systematic comparison of three mathematically related representations of the same MOS sensor response: global magnitude (M), relative sensor fingerprint (CLR), and their combination (LGR). Using the same classifier and validation partitions, this study investigates how their discriminative performance changes across compounds, concentration levels, measurement sessions, and real table olive samples. This approach provides experimental evidence that signal representation should be selected according to the analytical objective and the expected sources of variability, rather than solely on calibration accuracy.

## 2. Materials and Methods

### 2.1. Study Design and Experimental Units

The study comprised two connected experimental campaigns: a reference-compound campaign conducted from 31 August to 11 September 2026, and a table olive campaign conducted from 15 to 18 September 2026. The reference-compound experiment was designed to vary both compound class and nominal concentration under controlled conditions. The experimental unit was one prepared solution measured in one vessel. Three preparations were available for each compound × level condition and were distributed across sessions. Dilutions prepared on the same day shared the corresponding C1 stock and therefore should not be interpreted as 36 independent reagent batches.

For olives, each sample code corresponded to a different sealed commercial can. The usable cycles from each acquisition were aggregated before modeling and were never split between training and validation. The acquisition order was predefined before measurement, and C, DS, and DF positions were alternated across sessions so that category was not systematically associated with measurement day or position within a session. The supplier assigned the three quality categories before the electronic-nose study. Exact production-lot identifiers were not available to the author; consequently, the analysis was conducted at the individual can/category level and does not claim independent lot-to-lot validation. Table 1 summarizes the experimental design and evaluation roles.

The acquisition sequences reported in Table 2 and Table 3 were predefined, except for the final replacement C_1 acquisition, which was added after PATf in S4; chronological analyses use its actual acquisition date. During the olive campaign, category position changed across sessions, and all three calibration categories were represented in each session, reducing complete confounding between category and measurement day. The six reserve cans were designated before model fitting and were excluded from calibration, preprocessing fitting, and cross-validation. They are therefore described as a reserve set for within-campaign comparison rather than as an independent external validation set.

### 2.2. Electronic Nose and Measurement Principle

Measurements were performed with a NASYS S3+ electronic nose (Nano Sensor Systems S.r.l. (NASYS), Brescia, Italy) configured with two three-channel metal-oxide semiconductor (MOS) gas-sensor modules, providing six analytical channels identified as M1R1, M1R2, M1R3, M2R1, M2R2, and M2R3. The manufacturer describes the S3+ as a customizable gas-sensor instrument based on arrays of metal-oxide nanowires or nanostructures, and independent studies describe the platform as a multichannel MOS system with a controlled sensing chamber and dynamic sampling circuit [7,21]. The specific sensing-layer composition of the six channels in the customized configuration used here was not available to the author; therefore, no oxide-specific or molecule-specific selectivity was assigned to individual channels. Sensor heaters were operated at 500 °C, the nominal sampling flow was 190–200 mL/min, and resistance was acquired at 1 Hz.

In all experiments, the MOS array measured the headspace generated in a closed sampling vessel, rather than the liquid or solid samples directly. The preparation and sampling procedures for reference solutions and olive samples are detailed in Section 2.3 and Section 2.4, respectively.

The sensing-layer compositions of the six channels are proprietary and were not available; the manufacturer describes them as nanostructured metal-oxide thick-film sensors [7,21]. The analysis therefore treats the six channels as cross-sensitive members of one array and does not attribute chemical selectivity to individual channels.

Figure 1 shows the S3+ measurement setup used in the study, including the 190 mL sample vessel and the thermostatic water bath (Baño Cata, Bunsen, Humanes de Madrid, Spain) used to condition the headspace at 25 °C. Figure 2 summarizes the three mathematically linked signal representations evaluated from the same six-channel MOS response. Figure 3 shows a recorded acquisition cycle.

### 2.3. Reference Compounds

Ethanol (ETA), acetone (ONA), and acetic acid (ACE) were obtained from PanReac AppliChem, Panreac Química S.L.U. (Castellar del Vallès, Barcelona, Spain). Hexanal (HEX) was obtained from Sigma-Aldrich, Merck Life Science S.L.U. (Madrid, Spain). Ultrapure water was produced in-house using a Milli-Q water-purification system (Millipore/Merck, Molsheim, France). The four reference compounds were prepared in ultrapure water at the nominal liquid-phase levels shown in Table 4. They represent four different chemical families—an alcohol, a ketone, a carboxylic acid, and an aldehyde—and provide chemically distinct challenges to the cross-sensitive MOS array. Ethanol and acetic acid are relevant to fermentation-related headspaces, whereas acetone and hexanal extend the response space to additional functional groups [7,15,16]. These solutions were used as controlled instrumental challenges and were not intended to reproduce the volatile composition of table olive samples.

The concentration range spanned approximately one order of magnitude for each compound. Concentrations refer to the liquid phase; gas-phase concentrations or partial pressures at the sensor array were not measured, and the nominal levels should therefore not be interpreted as equivalent molar doses across compounds. Ethanol C1 was prepared from a 10% *v*/*v* stock solution, and acetone C1 from a 1% *v*/*v* stock solution; C2 and C3 were obtained by diluting the corresponding C1 solution. Each prepared solution was measured once using four acquisition cycles. The acquisition recorded on 7 September as ETA_C3 was subsequently verified to have been prepared at the ETA_C1 concentration and was relabeled accordingly; ETA_C3 was then prepared at the correct concentration and measured on 9 September. One ultrapure-water blank was included in each session, giving 36 reference-solution acquisitions and six water controls.

### 2.4. Table Olives and Supplier-Defined Quality Categories

Sealed commercial cans of Californian-style oxidized black table olives of the Cacereña variety and the same commercial caliber were supplied by Olives And Pickles, S.L. (Almendralejo, Badajoz, Spain). Before the electronic-nose study, the supplier had assigned each can to one of three quality categories used as sample metadata: conforming (C), mild barnyard-like off-odor (DS), or strong barnyard-like off-odor (DF). The off-odor was described by the supplier as a recurrent production-quality issue characterized by manure-like, rancid, and deteriorated-leather notes. Exact production-lot identifiers were not available to the author; therefore, the study does not infer how many independent production lots were represented. The cans remained sealed until preparation for electronic-nose measurement, and storage history before receipt was not independently audited.

No new human sensory assessment was conducted as part of this study, and no individual assessor data were collected or analyzed. The supplier-provided C/DS/DF categories were treated as pre-existing sample metadata rather than as sensory outcomes generated by the present experiment. The definitive olive dataset comprised 24 different cans: 15 calibration cans (five per category), six reserve cans (two per category) excluded from calibration, preprocessing fitting, and cross-validation, and three coded unknown cans whose supplier-assigned categories remained concealed from the analyst until predictions had been recorded.

Each sealed can was opened immediately before analysis. Four whole olives of the same commercial caliber were taken with clean tweezers and placed, together with 10.0 mL of the brine from the same can, in a 190 mL glass vessel (laboratory grade) fitted with a cap carrying an inlet and an outlet port. The closed vessel was placed in a thermostatic water bath at 25 °C for 5 min (Figure 1). The vessel outlet was then connected to the sample inlet of the S3+ through a short PTFE line and the vessel inlet received air filtered through a disposable granular activated-carbon cartridge supplied by the instrument manufacturer (NASYS, Brescia, Italy), so that the headspace above the olives and brine was carried to the sensor chamber by the instrument pump at 190–200 mL/min during the 45 s exposure phase of each cycle; the olives themselves and the brine never entered the instrument. Four cycles (100 s reference air, 45 s sample, 300 s cleaning) were recorded without reopening the vessel, taking about 30 min per can. The vessel was single-use and was discarded with its contents after each can; the next sample was prepared during the cleaning phase. Water blanks (AGU) and the ethanol–acetone verification mixture (PAT) were presented in the same vessel, volume and conditioning procedure. The acquisition order within each session is given in Table 3. The 5 min conditioning period at 25 °C was selected as a standardized pre-measurement interval, not as a demonstrated headspace equilibrium time. This practical duration corresponds to the 300 s cleaning/recovery interval between consecutive sample exposures in the acquisition protocol, avoiding longer analysis times. The same conditioning conditions were applied to all samples and controls. Headspace equilibrium was not independently verified. The use of intact fruit with its own brine follows previously reported headspace-presentation approaches [4,22].

### 2.5. Acquisition Sequence and Controls

All reference solutions, verification standards, and olive samples were conditioned and analyzed at 25 °C. This temperature refers to sample/headspace conditioning and is distinct from the MOS sensor-heater temperature (500 °C) and from the internal chamber-state temperature recorded during operation. Before the first measurement, the instrument was stabilized for 60 min under activated-carbon-filtered reference air. Each acquisition comprised four complete cycles: 100 s of activated-carbon-filtered reference air, 45 s of sample exposure, and 300 s of filtered-air cleaning/recovery, at approximately 190–200 mL/min. Cycle 1 was treated as a conditioning cycle and excluded from quantitative analysis. Among cycles 2–4, only complete cycles containing the full reference-air, sample-exposure, and cleaning/recovery sequence for all six channels were averaged to obtain one observation per physical sample; incomplete acquisition cycles were retained in the raw archive but excluded from averaging. Chamber temperature and relative humidity were recorded for each acquisition. Measurements performed outside the prevailing session temperature regime were identified and reported without applying a temperature correction.

During the olive campaign, ultrapure water (AGU) was measured at the start of each session, and a fixed ethanol–acetone verification mixture (PAT) was measured at both the start (PATi) and end (PATf) of each session. PAT contained 0.0100% *v*/*v* ethanol and 0.00333% *v*/*v* acetone. A single 500 mL batch was prepared, immediately divided into closed single-use aliquots, stored at 4 ± 2 °C, and brought to 25 °C before measurement. PAT was used to monitor within-campaign variation in instrument response to a nominally constant chemical stimulus and was not used to train the olive classifier. AGU and PAT measurements were excluded from all olive-class models.

### 2.6. Signal Representation: LGR, M, and CLR

Electronic-nose analysis conventionally separates signal preprocessing and feature construction from subsequent dimensionality reduction, prediction, and validation; logarithmic and relative response transformations and array normalization are established parts of this framework [17,18,19]. For each channel i and cycle, R_0,*i*_ was the mean of the final five records in the reference-air phase and R*_f,i_* was the mean of the final five records in the sample phase. Conductance is G = 1/R. The primary per-channel variable was the natural log-conductance ratio, abbreviated LGR:

LGR_i_ = ln(G*_i_*/G_0,_*_i_*) = ln(R_0,_*_i_*/R_f,*i*_)

The six-channel LGR vector was then decomposed into a common magnitude term and a relative profile. Global magnitude M was defined as the arithmetic mean of the six LGR values:

M = (1/6) Σ*_i_* LGR*_i_*

The centered log-ratio profile was obtained by subtracting this mean from each channel:

CLR*_i_* = LGR*_i_* − M, with   Σ*_i_* CLR*_i_* = 0

The decomposition is exact:

LGR*_i_* = M + CLR*_i_*

M therefore describes how strongly the six-channel array responds overall, whereas CLR describes how the response is distributed among channels after removing the common log-scale magnitude. LGR retains both components. The centering operation is mathematically analogous to Aitchison’s centered log-ratio transformation [20]: for positive conductance ratios, it is equivalent to taking the logarithm of each channel ratio divided by the geometric mean of the six ratios. Here, CLR is used as a symmetric transformation of sensor-response ratios and does not imply that sensor channels represent chemical proportions. Likewise, M is neither a chemical concentration nor a measure of perceived sensory intensity. Because the six CLR coordinates sum to zero, they contain at most five independent degrees of freedom; regularized LDA was therefore used rather than assuming a full-rank unregularized covariance matrix. Centering removes a common multiplicative factor in G/G_0_ exactly, but concentration changes need not affect all sensors proportionally; therefore, concentration-related information can remain in CLR. The three principal representations are summarized in Table 5.

As an additional reference in the compound experiment, an 18-variable block combining R_f_, R_0_ − R_f_, and (R_0_ − R_f_)/R_0_ was evaluated. It was not interpreted as a pure composition descriptor because it mixes absolute and baseline-normalized information. No water-blank subtraction was applied to the principal comparisons.

### 2.7. Reference-Compound Classification and Validation

To compare signal representations under equivalent conditions, the same classifier—linear discriminant analysis (LDA)—was applied to M, CLR, LGR, and the 18-variable benchmark representation. Variables were standardized using parameters estimated exclusively from the training data within each validation partition. LDA was implemented with the lsqr solver and automatic covariance shrinkage (shrinkage = ‘auto’ in scikit-learn 1.9.0). The fixed seed 20260809 was used only to reproduce partitions requiring deterministic group assignment. AGU measurements served as controls and were not included as a fifth reference class.

Four complementary validation schemes were used, each addressing a different source of variation. First, grouped condition cross-validation kept together the three preparations belonging to each compound × level condition and summarized one decision per condition (12 decisions). Second, leave-one-session-out validation held out each of the six measurement sessions in turn, yielding 36 preparation-level predictions. Third, leave-one-level-out validation trained on two nominal concentration levels and predicted the third across all four compounds, again yielding 36 predictions. Finally, a temporal S6 holdout trained on S1–S5 and predicted the six preparations measured in S6. These partitions are deliberately not interchangeable; they challenge transfer across compound × level conditions, measurement sessions, concentration levels, and a later session, respectively. This structure follows the general principle that validation partitions should respect temporal and hierarchical dependence [23].

### 2.8. Olive Classification, Matched Representation Comparison, and Model Selection

The primary applied contrast was conforming C versus off-odor D = DS + DF. To isolate the effect of signal representation, M, CLR, and LGR were compared using the same regularized LDA classifier and the same five can-level cross-validation folds. Preprocessing and standardization were fitted using training data only. The fixed seed 20260809 was used solely to reproduce the fold assignment, and all three representations were evaluated on exactly the same partitions. This matched design was essential because changing both the representation and the classifier would prevent attribution of performance differences to signal representation alone. LDA with automatic covariance shrinkage was selected as a relatively parsimonious and interpretable supervised classifier for the small calibration dataset (15 cans and at most six variables). It avoids extensive hyperparameter tuning and is widely used in electronic-nose pattern analysis [18]. More flexible models would increase the risk of overfitting given the limited number of independent samples. Binary classification (C vs. off-odor) was selected because the intended screening decision is to distinguish conforming from defective samples, while DS and DF represent two severity levels of the same supplier-assigned defect. Given the limited number of independent cans per category (*n* = 5), three-class classification was not attempted. DS and DF were therefore analyzed separately in the exploratory assessment but combined into a single off-odor class for supervised classification.

The six reserve cans had been assigned that role before model fitting and were excluded from calibration, preprocessing fitting, scaling, and cross-validation. They were subsequently used for within-campaign comparison of M, CLR, and LGR. Because reserve-set performance contributed to the assessment of signal representation, these six cans are not presented as an unbiased external test set for a finally selected representation. LGR was subsequently retained because it preserves both global magnitude and relative sensor-profile information. Within LGR, LDA and partial least-squares discriminant analysis (PLS-DA) were compared by grouped cross-validation balanced accuracy, with LDA preferred for parsimony and direct interpretability when performance was tied. For PLS-DA, the number of latent variables was selected independently within the training data of each outer partition rather than fixed globally. Candidate models with one to six latent variables, subject to the rank and size of the corresponding training set, were evaluated by internal cross-validation, and the smallest number of latent variables was preferred in the event of equal performance. Consequently, no single latent-variable count applies to all outer validation partitions.

Three additional cans were measured under coded identities, with their supplier-assigned categories concealed from the analyst until predictions had been recorded. These samples were used as a coded-prediction workflow check rather than as a statistically powered validation set. A retrospective chronological evaluation was also performed using only calibration cans ordered by their actual acquisition date: train on S1 and test on S2; train on S1–S2 and test on S3; and train on S1–S3 and test on S4. At each transition, scaling and model parameters were re-estimated using only the preceding sessions. For LGR, this chronological scheme was used to compare LDA and PLS-DA. In addition, as a post hoc representation-level sensitivity analysis, the identical chronological partitions were applied to M, CLR, and LGR using the same LDA classifier.

The study therefore distinguishes three evaluation roles: grouped cross-validation for calibration-set performance, a six-can reserve set for within-campaign representation/model comparison, and three coded unknowns for a concealed-category prediction demonstration. No reserve or coded sample was used for model fitting.

### 2.9. Metrics and Visualization

Balanced accuracy (BA), defined as the mean of class sensitivities, was the primary summary metric because the binary calibration task contained five C and ten D cans. Correct classification counts, class-specific sensitivities, and confusion matrices were reported alongside BA, and Matthews correlation coefficient (MCC) was used for binary comparisons. Given the small number of physical cans, a single prediction can materially affect the reported metrics; numerical differences among representations or models were therefore interpreted descriptively rather than as formal statistical evidence of superiority. For class sensitivities in the small olive sets, 95% Wilson score intervals were used as descriptive uncertainty bounds; no Wilson interval was assigned directly to BA.

For visualization of the olive dataset, principal component analysis (PCA) was fitted using only the 15 calibration cans, and reserve cans were projected into the fitted space without refitting either preprocessing or PCA. The main PCA used the six LGR channels. The final binary LDA discriminant score was also plotted. Because binary LDA contains only one discriminant dimension, the vertical position used to separate supplier categories graphically is a display offset and does not represent a second discriminant axis.

### 2.10. NECA ML Software Environment and Reproducibility

NECA ML is an in-house research software environment developed by Ramiro Sánchez Baltasar at CICYTEX for electronic-nose data processing and model evaluation. The analyses reported here were performed with NECA ML v0.9.19 (build B0.9.19, CICYTEX, Badajoz, Spain), implemented in Python 3.11.4 (Python Software Foundation, Wilmington, DE, USA) with NumPy 2.4.6, pandas 3.0.5 and scikit-learn 1.9.0. The software preserves the experimental hierarchy from acquisition to prediction: files remain linked to their physical-sample roles, cycles from the same acquisition remain within the same physical sample, preprocessing parameters are fitted using training data only, and calibration, reserve, coded, blank, and verification-standard roles remain distinct. NECA ML integrates signal inspection, feature calculation, M/CLR/LGR representation, exploratory PCA, classification, grouped and chronological validation, reserve-set prediction, confusion matrices, and scientific export.

The primary analyses used a frozen internal NECA ML analysis build. Feature calculations, validation partitions, and LDA predictions were subsequently cross-checked by rerunning the exported feature matrices in a separate Python/scikit-learn environment. NECA ML is not presented as an open-source software contribution. Reproducibility of the reported scientific comparisons instead relies on the documented signal equations, model settings, validation logic, feature matrices, and exported predictions, which allow the statistical analyses to be reproduced independently of the graphical application. Figure 4 summarizes the workflow.

## 3. Results

### 3.1. Response Magnitude and Concentration Dependence of Relative Profiles

Global response magnitude M increased with the nominal levels of ethanol, acetone, and hexanal. The descriptive slopes per concentration decade were 0.689, 0.709, and 0.595, with R^2^ values of 0.924, 0.915, and 0.941, respectively (Figure 5). Acetic acid behaved differently, with a slope of 0.004 and an R^2^ of 0.001 under the tested concentrations and protocol. This flat response may reflect aqueous headspace partitioning and the cross-sensitive response of the MOS array, but gas-phase concentrations were not measured and no mechanistic attribution is made. The water blank was not signal-free. The S5 water blank had been flagged for repeat in the contemporaneous quality-control record and was excluded from the descriptive blank summary; the specific reason for the repeat flag was not retained in the available metadata. Across the remaining five sessions, the mean water M was 0.252 ± 0.063. The practical implications of this near-flat acetic acid response are discussed in Section 4.2.

CLR profiles preserved differences among compounds but also changed with concentration (Figure 6). For ethanol, acetone, and hexanal, between-channel contrast tended to compress as the nominal level decreased. Acetic acid profiles were much more similar across levels. Thus, centering removed the common magnitude term but did not make the sensor fingerprint concentration-independent.

### 3.2. Reference Compounds: The Preferred Representation Depends on the Validation Question

With the same LDA classifier, M consistently underperformed the multichannel representations for reference-solution class (Table 6; Figure 7). CLR equaled or exceeded LGR in the three principal cross-validation schemes, whereas the 18-variable block achieved the highest leave-one-session-out BA but did not retain that advantage when the concentration level was excluded. The complete balanced accuracy values for all four evaluation scenarios are reported in Table 6.

With CLR and leave-one-session-out validation, the four errors were the three HEX_C3 preparations and ETA_C3 from S6; all were assigned to the ACE reference class. The errors therefore concentrated in low-response conditions. Because water was not a model class, assignment to ACE cannot be interpreted as demonstrated molecular identification or as separation of acetic acid from the water blank. In the S6 holdout, CLR and LGR each classified five of six preparations correctly, with accuracy 0.833 and BA 0.750, because the S6 class distribution was uneven.

### 3.3. Instrument-State Controls During the Olive Campaign

The response of PAT at session start increased from M = 0.4246 in S1 to 0.5510 in S4, a 29.8% increase (Figure 8a). Start-to-end differences within sessions did not have a consistent sign. PAT_2_r2 contained two complete post-conditioning cycles available for averaging; the remaining PAT acquisitions contained three. The water blank also changed by channel across sessions (Figure 8b). These controls were kept separate from olive categories and were not used to train the screening model. The PAT change is therefore described as instrument-state variation rather than attributed exclusively to sensor drift.

The recorded chamber temperature showed two regimes, approximately 48 and 64 °C; the cause of this shift could not be established from the retained acquisition metadata. Among calibration cans, DS_1 and DF_4 remained in the high regime and DS_4 transitioned between regimes; reserve C_7 also transitioned, and coded UNK_1 was measured in the high regime. These observations were retained rather than retrospectively corrected. Importantly, the category ordering of session-wise mean M remained C > DS > DF in all four sessions despite the changing PAT response: 0.865/0.672/0.612 in S1, 0.875/0.818/0.677 in S2, 0.889/0.764/0.691 in S3, and 0.911/0.882/0.781 in S4 (C/DS/DF, respectively). The chamber temperature and relative humidity recorded during acquisition did not differ significantly among supplier categories (Kruskal–Wallis, *p* = 0.80 and *p* = 0.97; *n* = 21 cans) and were not significantly correlated with M (Spearman ρ = −0.09 and 0.01, respectively).

### 3.4. Olive Structure: A Strong Magnitude Gradient but Incomplete Separation of Supplier-Defined Categories

Figure 3b shows representative cycle-2 sensor traces from one can per category (C, DS and DF). The signals illustrate the MOS response to olive headspace, with the largest relative-resistance decrease observed for the selected conforming can and the smallest for the selected strong-off-odor can, consistent with the overall magnitude gradient reported below.

Figure 9 provides an unsupervised exploration of the olive sensor responses, whereas Figure 10 evaluates their supervised classification performance using different feature representations. PCA fitted on the 15 calibration cans explained 88.5% and 10.9% of calibration variance with PC1 and PC2, respectively (Figure 9). Reserve cans were projected without refitting the axes. C occupied mainly the positive PC1 region and DF the negative region, while DS was more dispersed and overlapped both. The plot therefore shows a broad response gradient rather than three isolated categories.

The same gradient was visible in M. Across the 21 non-coded cans, the mean ± standard deviation M was 0.888 ± 0.050 for C, 0.770 ± 0.085 for DS, and 0.677 ± 0.070 for DF. The observed ranges were 0.833–0.954 for C and 0.599–0.781 for DF, whereas DS spanned the two response regions. Because the supplier labels describe off-odor severity rather than total volatile load, M should be interpreted as the overall MOS-array response, not as a sensory-intensity scale.

### 3.5. Same LDA, Different Representations: Calibration and Reserve-Set Performance Diverge

Figure 10a is the key matched comparison of balanced accuracy in olives; Figure 10b shows the corresponding reserve-set confusion matrices. The classifier, grouped folds, physical samples, and binary endpoint were kept fixed; only the signal representation changed. M produced the highest grouped-CV BA (0.900), with all five C cans correctly classified but two D cans assigned to C. CLR and LGR each produced BA of 0.850 in grouped CV, with one error in each direction. If only calibration-set cross-validation were considered, the single-variable magnitude representation would appear preferable.

The reserve set changed that ranking. M correctly classified four of six cans (BA 0.750), CLR five of six (BA 0.875), and LGR all six (BA 1.000). Because these six cans were used to compare representations, the result is a within-campaign model-development comparison rather than independent external validation. Within this small reserve set, the ranking suggests that retaining both global magnitude and relative sensor-profile information was associated with a higher correct classification count in this reserve set, but the difference between CLR and LGR corresponds to a single can and is interpreted descriptively. The matched numerical comparison is summarized in Table 7.

Unlike the exploratory PCA shown in Figure 9, this figure compares the supervised classification performance of LDA using M, CLR, and LGR under matched validation conditions.

The primary screening endpoint was defined a priori as conforming (C) versus off-odor (D = DS + DF) and is retained throughout the reported model comparison. DS and DF are shown separately in plots and post hoc scope analyses to represent the supplier-assigned mild and strong off-odor categories; no validated three-class grading of defect intensity is claimed.

A retrospective temporal sensitivity analysis provided an additional representation-level comparison without using the six reserve cans. Using the same LDA classifier and a train-earlier/test-later scheme, M correctly classified 7/10 cans (BA 0.750; MCC 0.535), whereas both CLR and LGR classified 9/10 (BA 0.917; MCC 0.816; Table S3). CLR and LGR made the same single error (DS_5), whereas M additionally misclassified DS_3 and DF_5. Thus, the chronological analysis supports the value of retaining the multichannel response pattern over global magnitude alone, but does not distinguish between CLR and LGR.

### 3.6. Final LGR-LDA Model, Reserve Cans, Chronological Evaluation, and Coded Prediction

The final LGR-LDA score plot is shown in Figure 11a. DS and DF share the modeled D class; their separation from one another is not the objective of the binary model. All six reserve cans fell on the correct side of the forced-class boundary, with DS_6 and DS_7 closest to it. Figure 11b places the same non-coded samples in the magnitude dimension and illustrates that magnitude is informative but does not by itself account for the reserve-set result.

In grouped CV, the final LGR-LDA model produced 13/15 correct predictions, BA 0.850, and MCC 0.700. On the six reserve cans, it predicted 2/2 C and 4/4 D correctly (BA 1.000, MCC 1.000). PLS-DA tied LDA in grouped CV but predicted 5/6 reserve cans correctly, assigning DS_6 to C (Table 8). For LGR, the retrospective chronological evaluation favored LDA: 9/10 correct (BA 0.917; MCC 0.816) versus 8/10 for PLS-DA; the LDA error was DS_5. The matched chronological comparison among M, CLR, and LGR using the fixed LDA classifier is reported separately in Table S3.

The three coded cans received forced classes D, D, and C from both LDA and PLS-DA, concordant with the concealed supplier key. Their category identities were not available to the analyst until after predictions had been recorded. With *n* = 3, this is reported as a coded-prediction workflow demonstration rather than a validation estimate.

### 3.7. Post Hoc Scope Checks

Two post hoc checks were used to define the limits of the screening results rather than to add new validation claims. First, one off-odor category was omitted from training at a time. When LDA was trained with C + DS and all seven DF cans were treated as an unseen D category, M, CLR, and LGR each classified 7/7 DF cans as D. When the model was trained with C + DF and the seven DS cans were treated as unseen D, the sensitivities were 3/7 for M and 4/7 for CLR and LGR. The missed mild-defect cans included DS_5, DS_6, and DS_7, whose magnitudes fall in the C response region. Thus, the strong category generalized more readily than the mild category; this check mitigates, but does not eliminate, concern that the classifier merely memorizes the represented categories. The main summary is shown in Table 9, with the detailed post hoc output in Table S2.

Second, sensitivity to the observed chamber-temperature regimes was examined by removing the three high or mixed calibration acquisitions (DS_1, DF_4, and DS_4) and repeating the reserve-set comparison. The LGR reserve result remained 6/6, and the M reserve result remained 4/6; the two LGR grouped-CV errors remained C_3 and DS_5, neither of which belonged to the high/mixed subset. This analysis does not identify the cause of the chamber-state shift, but it indicates that the main reserve-set contrast was not created by those three calibration acquisitions. Detailed results are provided in Table S1.

These analyses were performed after inspection of the main results and are reported as descriptive scope/sensitivity checks, not as independent validation. For the unseen-category analysis, each test set contains only D samples, so balanced accuracy is not defined.

## 4. Discussion

### 4.1. Why Separating Magnitude from Fingerprint Matters

The algebraic decomposition itself is not new mathematics. Logarithmic/relative response manipulation, array normalization, feature construction, and pattern recognition are established in electronic-nose analysis [17,18,19], and centered log-ratio methods are well established in compositional data analysis [20]. The contribution here is experimental and methodological: a common log-scale magnitude term and centered channel deviations are used to create matched M, CLR, and LGR views of the same physical MOS response, and these views are challenged with the same classifier and the same physical-sample partitions across reference compounds, sessions, concentration levels, and a food matrix. Accordingly, novelty is claimed for the matched experimental design and validation evidence—not for the individual transformations or the CLR algebra.

This distinction matters because a visually convincing separation can have different physical explanations. If all six channels move together, M may be sufficient. If channels change disproportionately, the relative profile contains additional information. CLR removes a common multiplicative factor in the original positive ratios, but it does not make a real sensor array concentration-invariant. The reference-compound experiment demonstrates that limitation directly: CLR was far more informative than M for the reference-compound class, yet its profile still changed as nominal concentration decreased.

### 4.2. What the Reference Compounds Reveal About Response Class, Concentration, and Instrument State

The reference-compound experiment supports the use of defined stimuli before interpreting a complex food matrix. With the classifier held fixed, CLR was markedly more informative than M for reference-solution class: BA increased from 0.500 to 0.833 for grouped conditions, from 0.556 to 0.889 in leave-one-session-out validation, and from 0.528 to 0.833 in leave-one-level-out validation. This shows that relative channel response carried class information that a single global magnitude could not represent. At the same time, the compression of CLR profiles at lower levels shows why ‘shape’ should not be treated as synonymous with composition. Cross-sensitive MOS channels respond through redox processes at their sensing surfaces, so dilution can change the relative fingerprint as well as global amplitude. The weak response to aqueous acetic acid has practical implications for the applicability of the method. Under the tested conditions, acetic acid produced little change in the MOS-array response, suggesting limited sensitivity to this compound compared with the other reference standards. However, this observation cannot be directly extrapolated to complex olive headspace or to the detection of acid-related defects, which were not evaluated in this study. The classification results for the barnyard-like off-odor remain empirical findings specific to the tested samples. Since feature transformations cannot recover information that was not captured by the sensors, this potential limitation applies to M, CLR, and LGR. Complementary analytical measurements may be required when acetic acid is a target compound.

The validation scenario changed the ranking. The 18-variable block performed best when sessions were held out but not when levels were held out. This is precisely why one maximum percentage should not be used as a universal statement of representation quality. A representation is useful relative to the variation that the model is expected to encounter. For routine screening, session controls such as water and a fixed chemical mixture are therefore not cosmetic additions; they document whether the instrument is being challenged under a similar response regime and help distinguish sample effects from instrument-state changes.

### 4.3. Olive Screening: Calibration Ranking and Transfer Behavior Are Not the Same Result

The olive experiment is informative because grouped-CV and reserve-set rankings differed, while the retrospective chronological analysis added a third perspective. M alone achieved BA 0.900 during grouped calibration cross-validation with only one variable, showing that global response magnitude carried substantial screening information. On the six reserve cans, however, M achieved BA 0.750, CLR 0.875, and LGR 1.000. In the train-earlier/test-later analysis, M correctly classified 7/10 cans (BA 0.750; MCC 0.535), whereas CLR and LGR each classified 9/10 (BA 0.917; MCC 0.816). The chronological result therefore does not establish an advantage of LGR over CLR; instead, it indicates that the relative multichannel response pattern contains transferable information that is lost when the array is reduced to global magnitude alone. The additional advantage of LGR appeared only in the small reserve set, where retaining magnitude together with the relative profile yielded 6/6 correct predictions compared with 5/6 for CLR; this one-can difference is descriptive rather than evidence of statistical superiority.

The matched-LDA design is important. If M, CLR, and LGR had been assessed with different algorithms, performance differences could be attributed either to signal representation or to model flexibility. Holding the classifier and partitions fixed makes the representation comparison interpretable. Taken together, the grouped-CV, chronological, and reserve-set results do not show that one representation is universally superior; rather, they show that the preferred representation can change with the type of variation the model is required to encounter. This supports selecting electronic-nose features according to the analytical question and intended transfer scenario rather than solely from the maximum calibration cross-validation score.

The supplier categories also clarify the role of DS and DF. The electronic-nose magnitude was highest in C and lowest in DF, so the MOS array does not reproduce an ordinal ‘defect intensity’ scale. A stronger supplier-assigned off-odor category need not produce a larger total MOS response because MOS materials weight the headspace differently from human olfaction. Exact production-lot identifiers were not available, so the present study cannot distinguish a category effect from all possible production-lot effects. The supplier reported that the barnyard-like defect had been observed recurrently in routine production, but recurrence across independent lots was not experimentally verified here. The non-zero response of the water blank also shows that M is sensitive to the overall headspace matrix, not only to odor-active defect compounds. Because humidity, ethanol, water activity, and brine composition were not independently matched across supplier categories, part of the C > DS > DF magnitude gradient could reflect these covarying matrix factors; the present data therefore do not assign the magnitude difference uniquely to the barnyard-like defect chemistry. Although all olive samples were analyzed under standardized conditions, differences in ethanol content, water activity, and brine composition among samples cannot be excluded. Therefore, the observed C > DS > DF gradient in M should be interpreted as a difference in the overall MOS-array response to the sample headspace, rather than as a direct measure of off-odor intensity or specific defect-related compounds.

### 4.4. Instrument-State Variation Did Not Fully Account for the Observed Category Gradient

Instrument state changed during the campaign, but the available checks argue against it being the sole driver of separation. All three supplier categories were measured in every session, and the ordering of session-wise mean M remained C > DS > DF, while the PAT response increased by 29.8%. Across the same chronological train-earlier/test-later partitions, both CLR and LGR retained 9/10 correct predictions, whereas M retained 7/10. The shared CLR/LGR error was DS_5, while M additionally missed DS_3 and DF_5. In addition, excluding the three calibration acquisitions recorded in the high or mixed chamber-temperature regime left the LGR reserve result at 6/6 and the M reserve result at 4/6. These observations do not imply that instrument-state variation is negligible; they argue more narrowly that the reported category pattern is not explained simply by one category being measured in a different session or thermal regime. Although the sensing-layer heater temperature remained constant, variations in chamber temperature and relative humidity may affect MOS baseline resistance and response amplitude [8]. The two calibration cans measured entirely under the higher-temperature regime (DS_1 and DF_4) showed M values within their respective category ranges, and excluding the affected acquisitions did not change the reserve-set classification results (Table S1). Given the limited number of affected cans, a reliable temperature correction model could not be established. Therefore, chamber conditions should be monitored and measurements outside the prevailing temperature regime should be repeated rather than corrected. The chronological comparison and thermal-regime check are supporting robustness analyses rather than prospective validation. The temporal comparison was specified retrospectively, the transition-specific test sets are small, and the thermal-regime analysis was performed after inspection of the main results. Accordingly, the chronological result should be interpreted as evidence that retaining the relative multichannel pattern was more robust than using global magnitude alone under the observed within-campaign session changes, not as proof of universal temporal stability or of LGR superiority over CLR. Continued measurement of water and a fixed chemical reference remains necessary because electronic-nose calibration can lose temporal validity as sensors and operating conditions change [13].

### 4.5. Limitations and the Next Experiment

The limited number of physical samples is an important limitation of this study. In the 15-can calibration set, changing one prediction changes balanced accuracy by 0.05 or 0.10 depending on its class; in the six-can reserve set, it changes balanced accuracy by 0.125 or 0.25. Such one-can effects limit the precision of the differences between representations. Nevertheless, the results demonstrate that different feature representations of the same sensor response (M, CLR, and LGR) lead to different classification outcomes, even when the same classifier and validation partitions are used. Although the small calibration and reserve sets do not support definitive conclusions about the superiority of any particular representation, the observed differences highlight the importance of feature selection according to the analytical objective and validation conditions. Furthermore, because all samples were analyzed within the same experimental campaign and independent production-lot information was unavailable, further validation with additional production lots and measurement sessions is needed to assess the generalizability of these findings.

The principal sampling limitation is that exact production-lot identifiers were not available to the author. Each code corresponds to a distinct can, but the number of independent production lots represented within C, DS, and DF cannot be established from the retained metadata. Consequently, the manuscript makes can-level and supplier-category claims only. A future prospective campaign should record lot identifiers explicitly, include several independently produced batches per state, distribute them across measurement days, and evaluate a model fixed before the new campaign begins.

A second limitation is that the nominal reference concentrations were defined in the liquid phase; the gas-phase dose reaching the sensors was not measured. Headspace equilibrium was not demonstrated thermodynamically; 5 min was the standardized conditioning time. The olive load was standardized by fruit count because all samples had the same supplied commercial caliber, but four fruits are not identical to a gravimetrically fixed mass. Finally, the observed PAT and chamber-temperature variation confirms that instrument state should continue to be monitored rather than retrospectively ‘corrected’ on the same small dataset used to judge performance. The reserve-set comparison is also imprecise because it contains only six cans; differences such as 4/6 versus 6/6 are therefore reported descriptively and are not interpreted as statistically demonstrated superiority. The effect of longer conditioning periods on sensor responses remains to be evaluated. Because the sensing-layer compositions are not known, the CLR profiles are interpreted as array fingerprints and not mechanistically.

The most direct next experiment for separating composition-related pattern changes from total headspace load would involve varying the headspace load of the same physical olive sample—through controlled sample mass or headspace dilution—while keeping identity, temperature, and humidity controlled. Tracking M and CLR within the same sample across that manipulation would test whether magnitude and relative fingerprint respond as predicted by the decomposition. Combined with prospectively recorded independent production lots, this would test generalization more directly than searching for increasingly complex algorithms on the same cans. The conclusions are limited to the S3+ six-channel array and the olive variety evaluated in this study. Although the M/CLR/LGR representations can be applied to other electronic-nose arrays, their performance and transferability to other instruments and olive varieties require independent validation.

## 5. Conclusions

A six-channel MOS response contains at least two conceptually different sources of discriminative information: how strongly the array responds and how that response is distributed among channels. Expressing the same response as M, CLR, and LGR makes that distinction explicit. In defined reference solutions, relative profiles carried substantially more reference-class information than magnitude alone, although CLR remained partly concentration-dependent. In supplier-classified table olives, M gave the highest grouped cross-validation BA, but the retrospective chronological comparison favored both multichannel representations over M (9/10 correct for CLR and LGR versus 7/10 for M). The six-can reserve set then differentiated CLR and LGR descriptively: LGR classified 6/6 cans correctly, compared with 5/6 for CLR and 4/6 for M. Because the reserve set informed representation assessment, this is not independent external validation, and the one-can difference between CLR and LGR should not be interpreted as statistical superiority. The three coded cans were concordant with the concealed supplier categories in the final LGR workflow. Overall, the study provides a proof of concept that magnitude and relative fingerprint should be evaluated as distinct but complementary information sources, and that feature selection should follow the analytical question and expected transfer conditions rather than calibration-set performance alone. Prospective validation with explicitly recorded independent production lots remains the necessary next step. These findings concern the S3+ six-channel array and one variety of Californian-style black olives; transfer to other instruments, olive varieties, and production lots remains untested.

## Figures and Tables

**Figure 1:**
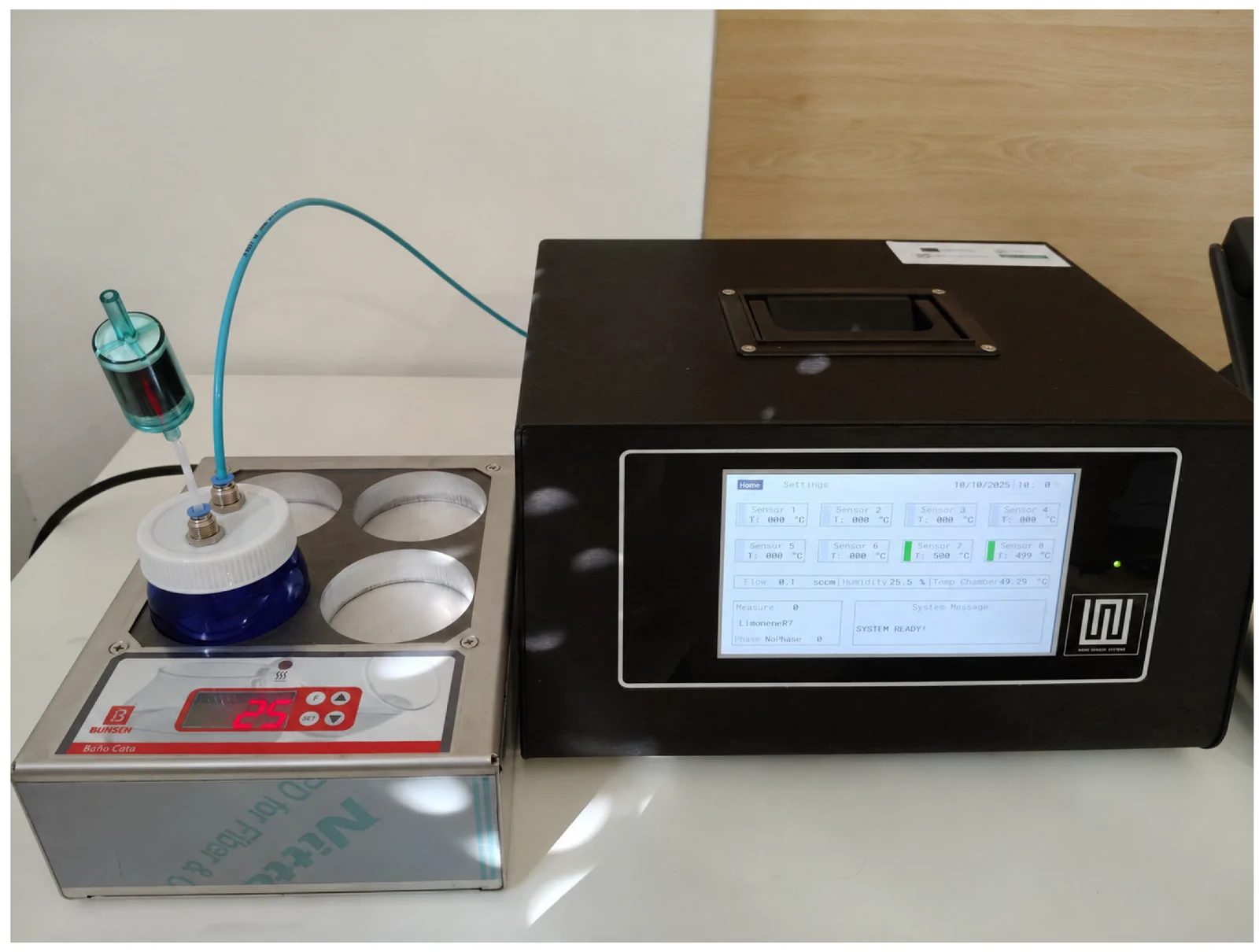
Experimental S3+ electronic-nose setup used in the study. A 190 mL vessel containing the test sample was conditioned in a thermostatic bath at 25 °C and connected to the NASYS S3+ through the sampling line. The photograph was taken in the author’s laboratory; the bath display shows the 25 °C setpoint used for sample/headspace conditioning. Left: thermostatic water bath with the 190 mL sample vessel and the activated-carbon filter cartridge; right: NASYS S3+ electronic nose.

**Figure 2:**
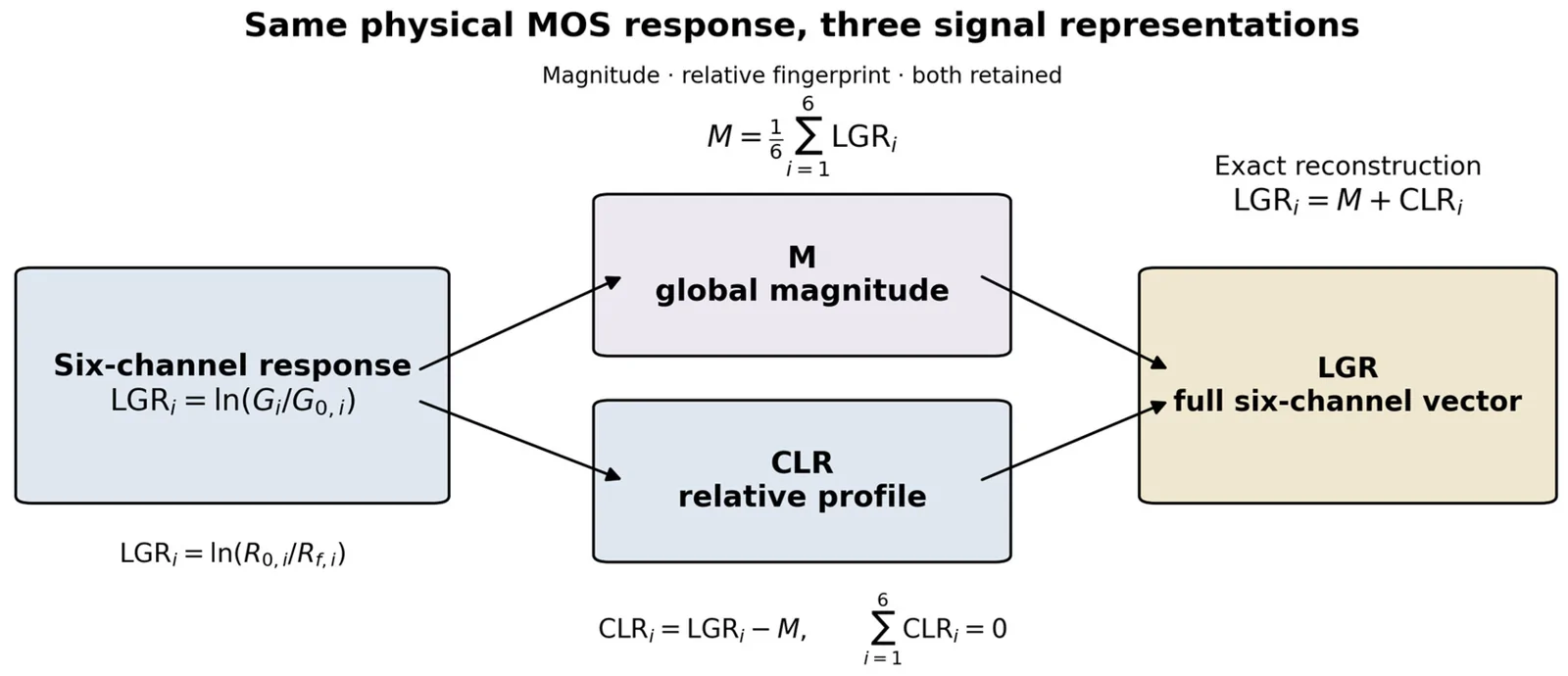
Same physical metal-oxide semiconductor (MOS) response expressed as three signal representations. LGR retains the six log-conductance-ratio channels, M is their mean and summarizes global response magnitude, and CLR centers each channel around M to describe the relative sensor profile. The decomposition is exact: LGR_i_ = M + CLR_i_. Arrows indicate the direction of the decomposition, from the six-channel response to M and CLR and their exact recombination into LGR; box colours only distinguish the three representations and carry no quantitative meaning.

**Figure 3:**
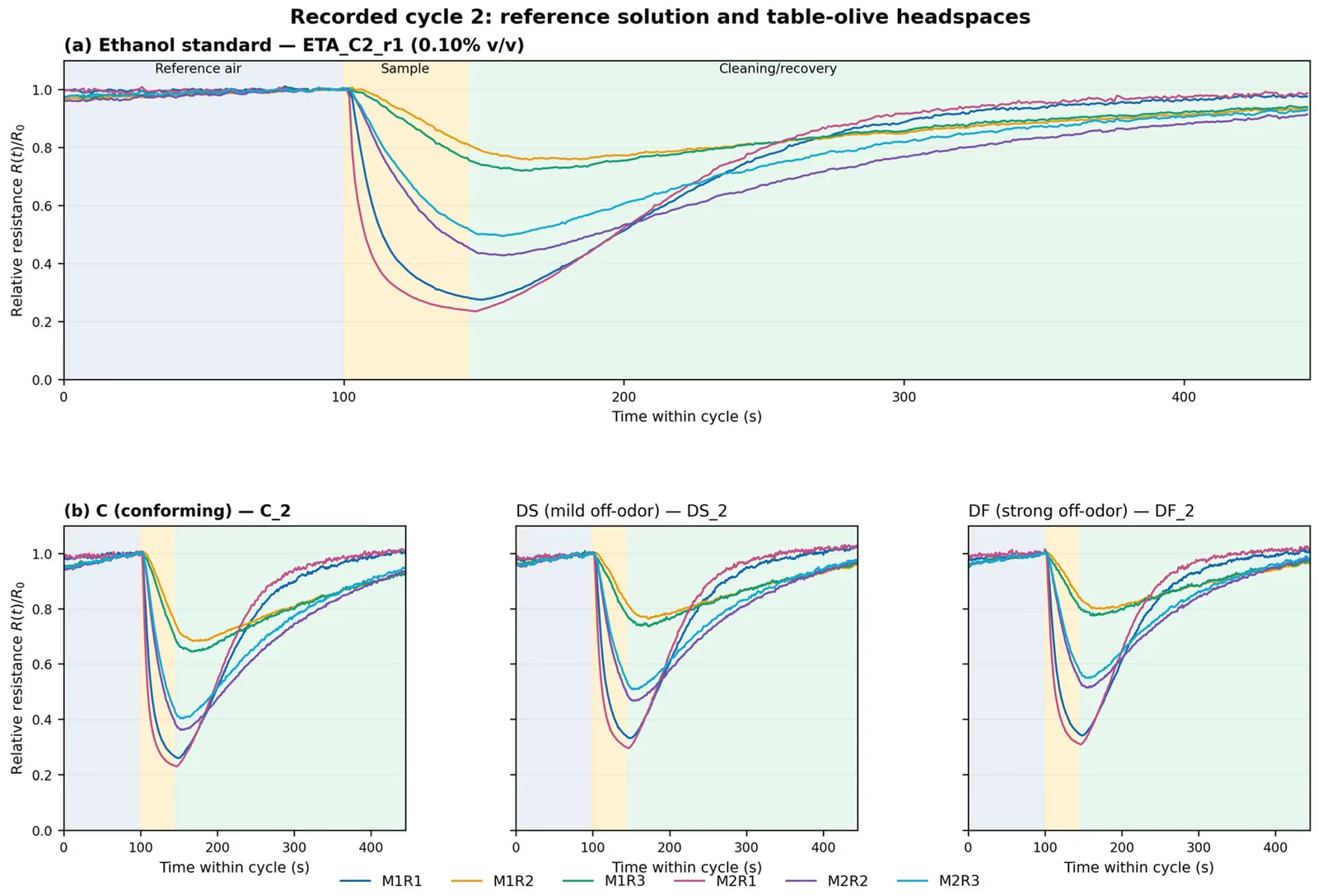
Recorded cycle 2 represented as relative resistance R(t)/R_0_ for the six MOS channels. (**a**) ETA_C2_r1 (ethanol 0.10% *v*/*v*). (**b**) One can of each supplier category measured in session S1 with the same protocol: C_2 (conforming), DS_2 (mild off-odor) and DF_2 (strong off-odor). Each cycle comprises 100 s of reference air, 45 s of sample exposure and 300 s of cleaning at 190–200 mL/min; the background shading marks the three phases (grey, reference air; yellow, sample exposure; green, cleaning/recovery), and line colours identify the six channels as in the legend; R_0_ is the mean of the last five records of the reference-air phase. The conforming can produced the deepest relative-resistance drop, consistent with the higher global magnitude M of the C category (Section 3.4).

**Figure 4:**
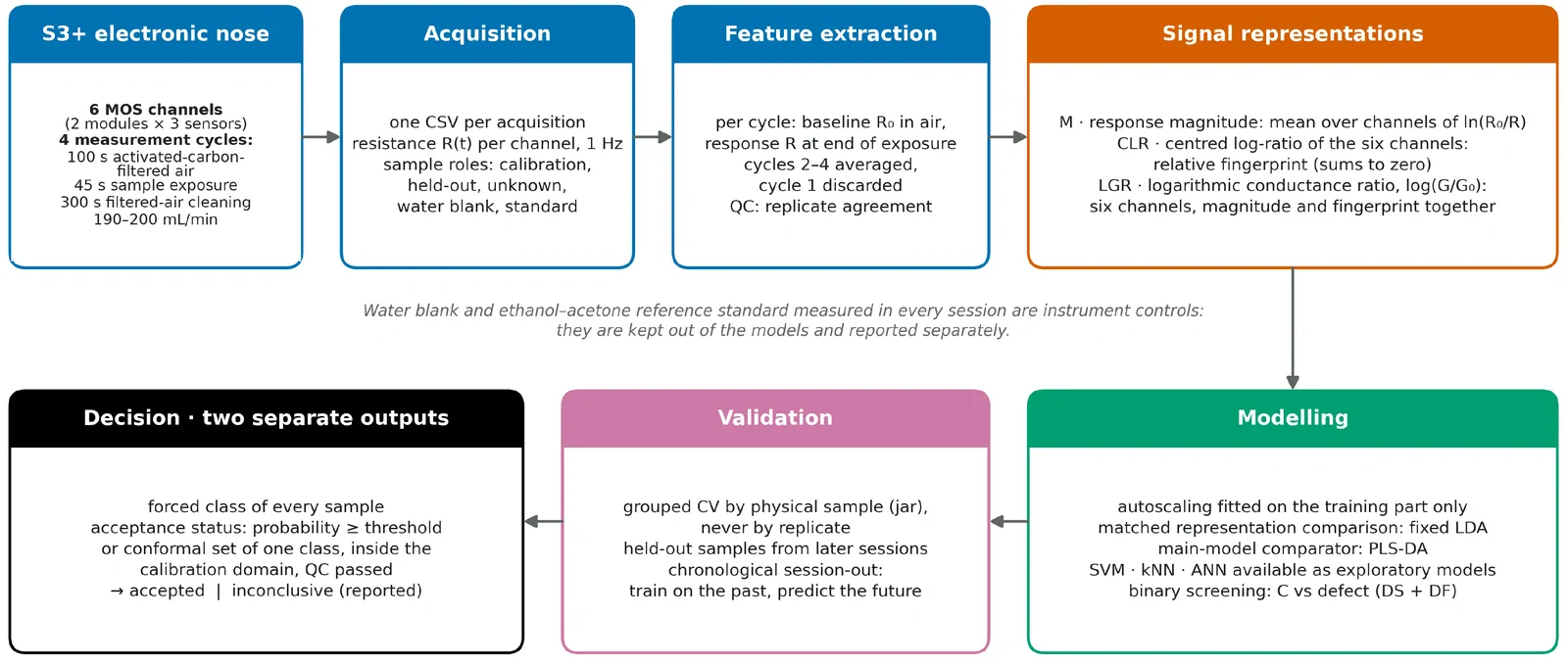
NECA ML analytical workflow used to keep experimental hierarchy, signal representation, validation, and prediction reporting separate. The S3+ acquisition consists of four cycles, each with 100 s of activated-carbon-filtered air, 45 s of sample exposure, and 300 s of filtered-air cleaning at 190–200 mL/min. Resistance traces from the six MOS channels are reduced to cycle-level features and expressed as M, CLR, or LGR. The matched signal-representation comparison uses a fixed LDA, while PLS-DA is used only as a secondary model comparator. Scaling is fitted on training data only. Validation is grouped by physical can and complemented by a reserve set, a chronological stress test, and coded-sample prediction. Water and ethanol-acetone controls are excluded from class models. The interface also contains an optional accepted/inconclusive decision module; however, that operational output is not used as a primary endpoint in this manuscript. Arrows indicate the flow of data from acquisition to decision. Box colours group the stages: blue, instrument, acquisition and feature extraction; orange, signal representations; green, modelling; pink, validation; black, decision outputs.

**Figure 5:**
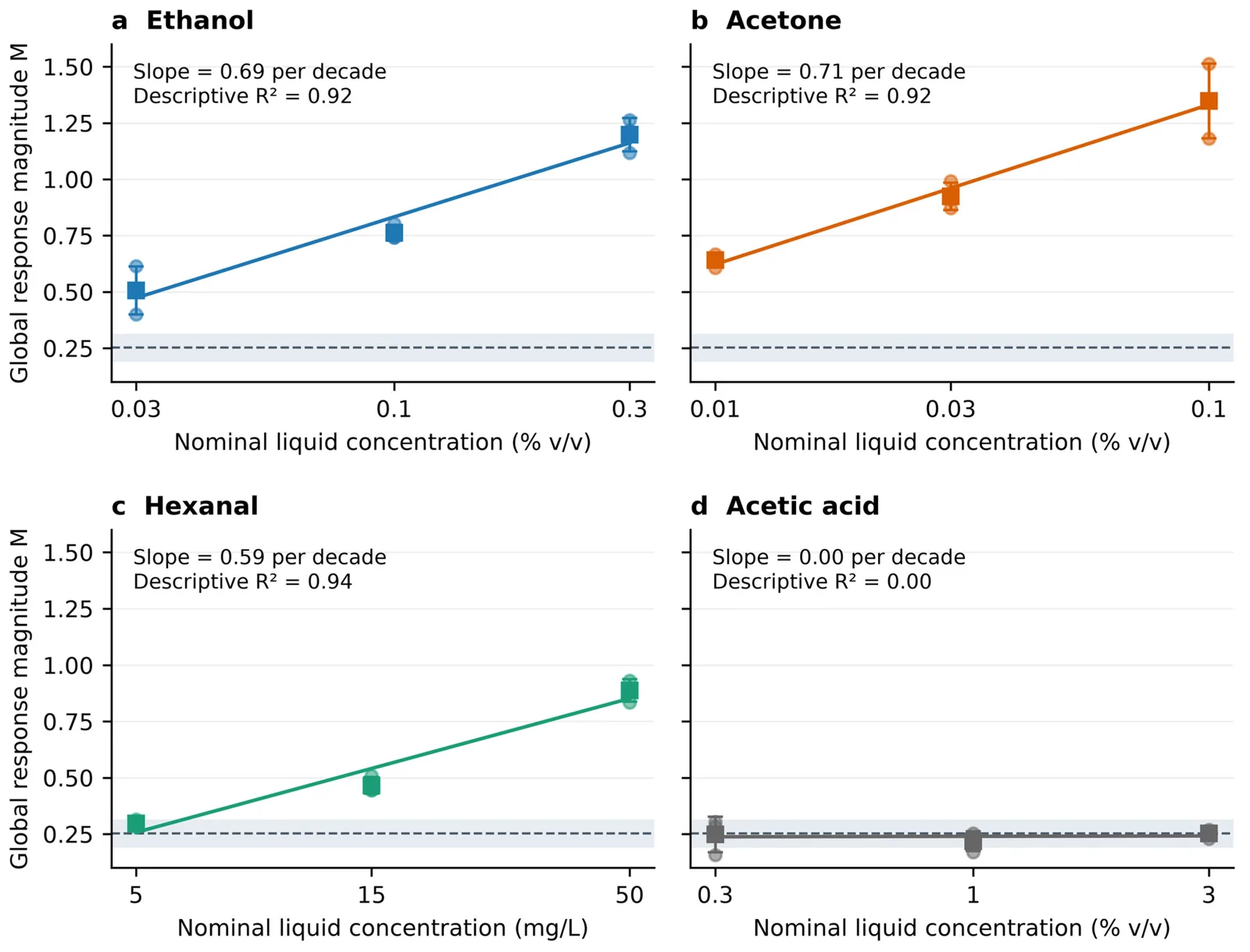
Global response magnitude M versus nominal liquid-phase concentration. Circles represent individual preparations; squares and error bars show mean ± standard deviation for the three preparations at each level. Lines are descriptive regressions of M against log10 nominal concentration. The dashed line and the gray band represents the mean and the mean ± standard deviation of five usable water blanks and is not a validated detection limit; colours only distinguish the four compounds. Concentration axes differ among compounds.

**Figure 6:**
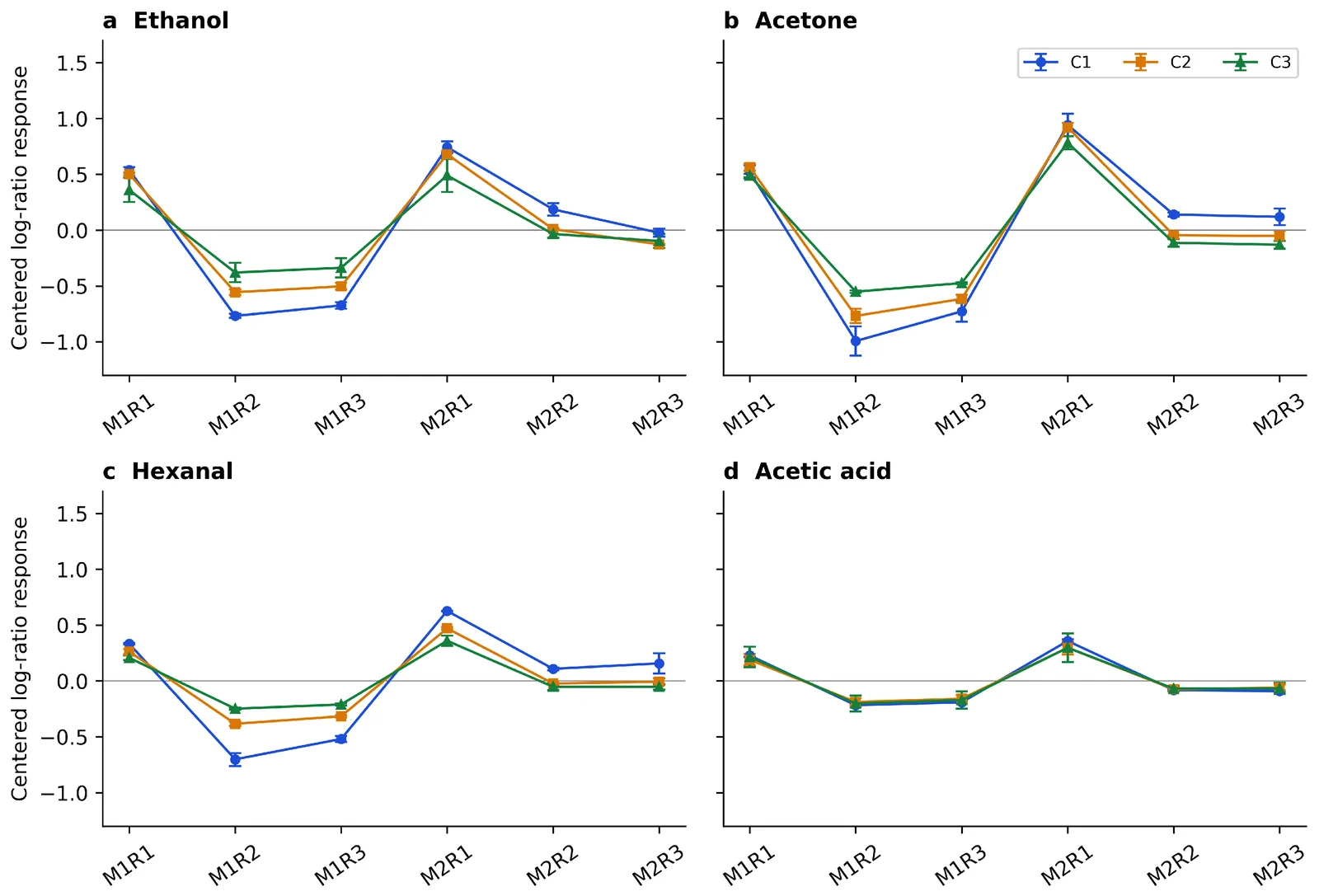
CLR profiles of the six MOS channels by compound and nominal level. C1, C2, and C3 correspond to the high, intermediate, and low concentrations in Table 4. Points and bars represent the mean ± standard deviation of three preparations measured in different sessions. Lines connect channels only to facilitate visual comparison and do not imply physical continuity among sensors. The horizontal grey line marks a centered log-ratio of zero, i.e., a channel equal to the array mean.

**Figure 7:**
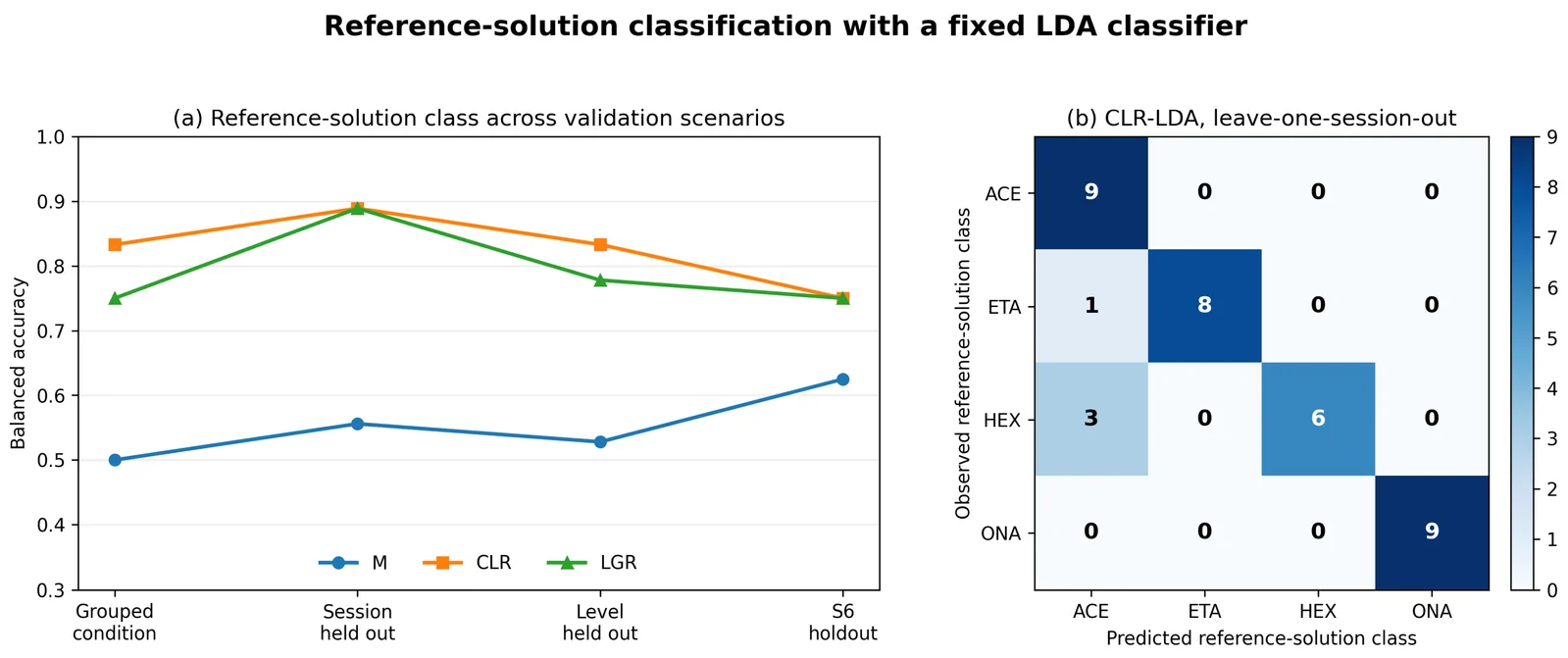
LDA validation of reference-solution class. (**a**) Comparison of M, CLR, and LGR across four validation scenarios. (**b**) Confusion matrix for CLR under leave-one-session-out validation, with nine preparations per reference compound. “ACE” denotes the acetic-acid solution class and does not imply molecular identification of the gas phase.

**Figure 8:**
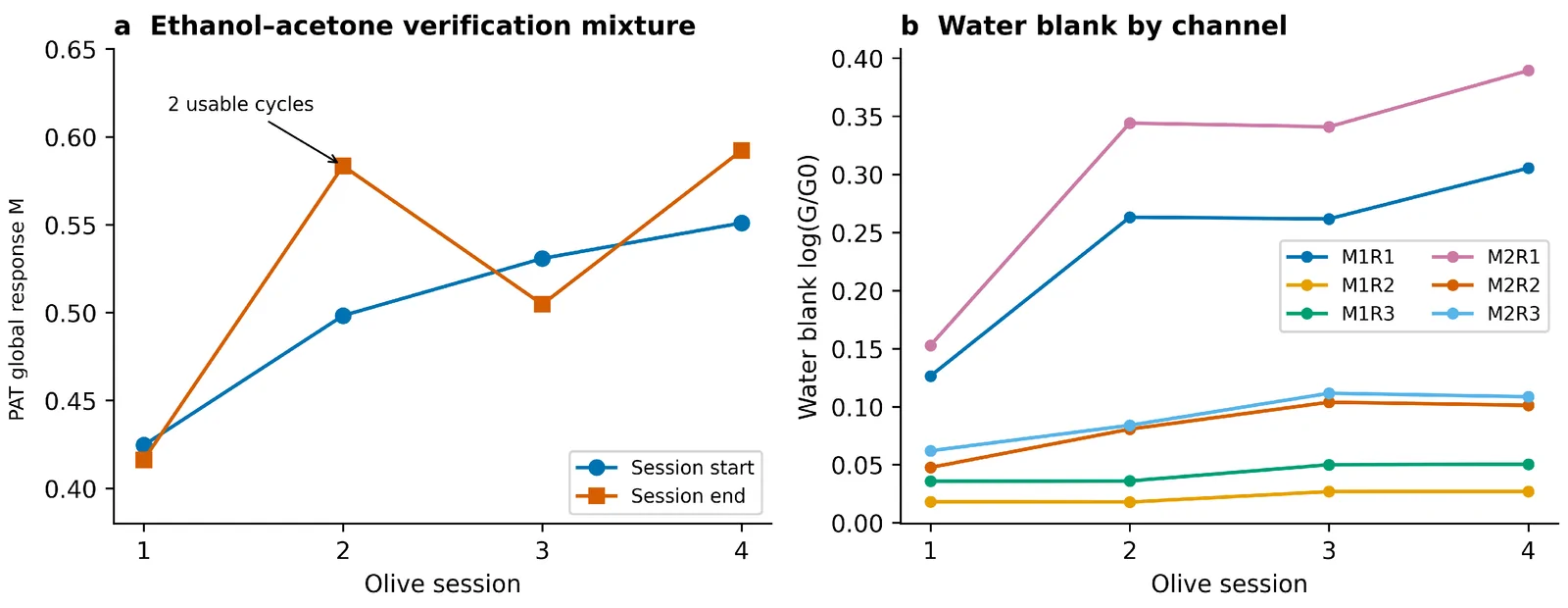
Controls during the olive campaign (15–18 September 2026). (**a**) Global magnitude M of the ethanol-acetone PAT mixture at the start and end of each session; the acquisition with two usable cycles is identified. (**b**) Water-blank LGR by channel. Connecting lines show temporal order only and are not fitted trends or validated control limits.

**Figure 9:**
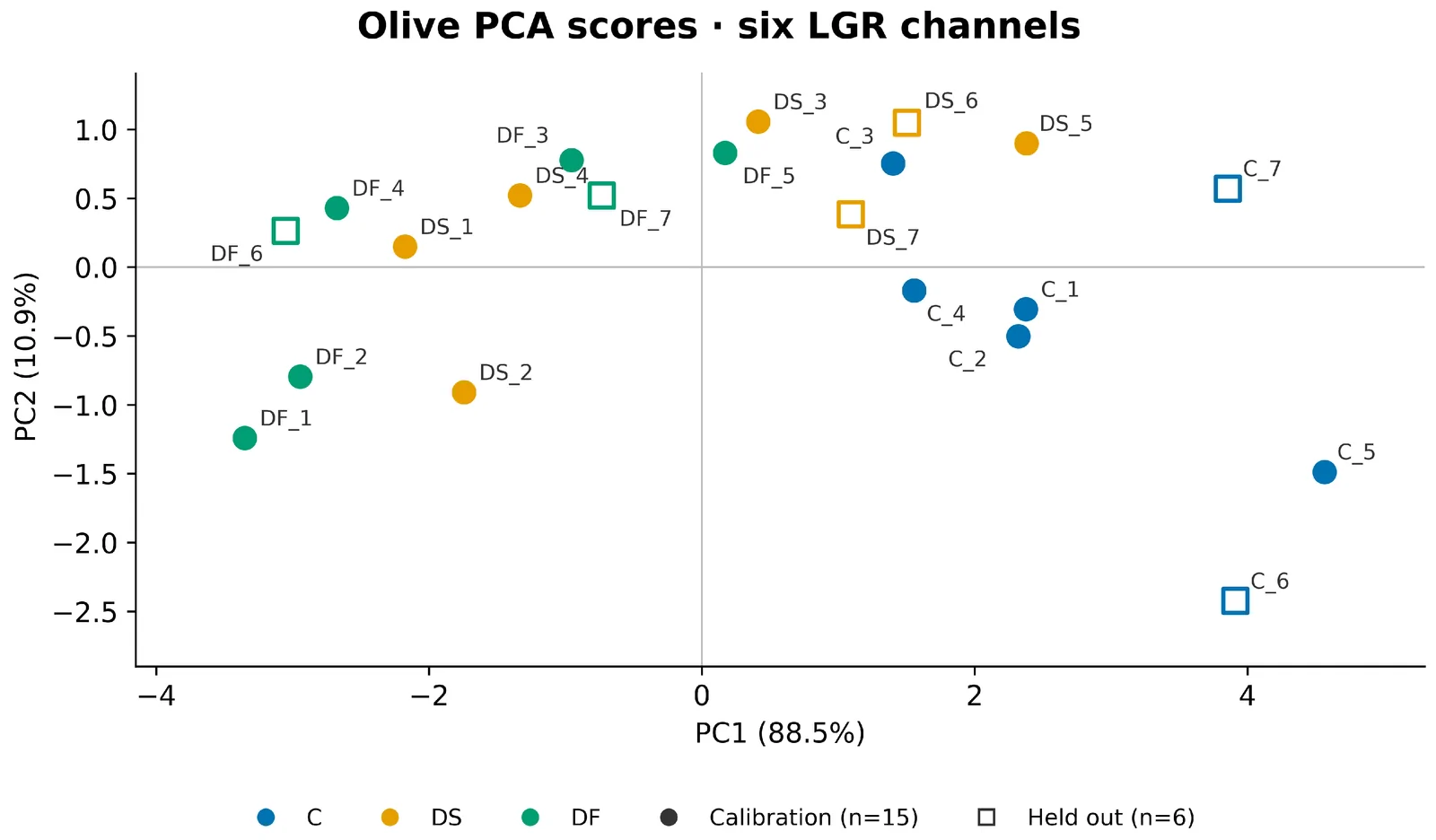
PCA scores for olives using the six LGR channels. Scaling and PCA axes were fitted only on the 15 calibration cans; six reserve cans were projected afterward. Filled circles: calibration; open squares: reserve. Colors identify the supplier-defined C, DS, and DF categories. PC1 and PC2 explain 88.5% and 10.9% of calibration variance, respectively. Category labels were used only for visualization and not for fitting the PCA model; supervised classification performance is reported in Figure 10.

**Figure 10:**
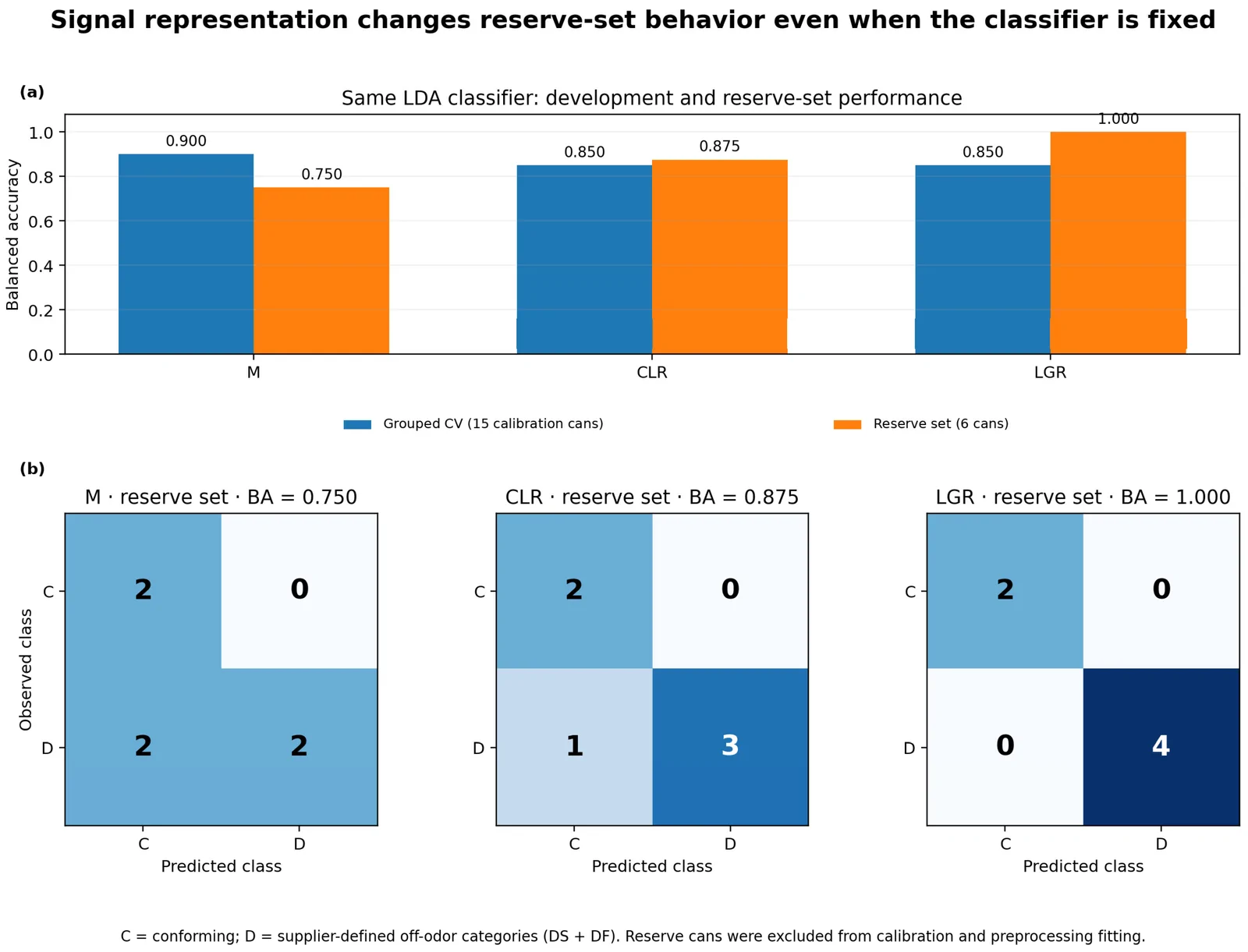
Same LDA classifier, different signal representations in the binary olive task. (**a**) The upper panel compares grouped cross-validation BA on the 15 calibration cans with BA on the six-can reserve set. (**b**) The lower panels show reserve-set confusion matrices for M, CLR, and LGR. C = conforming; D = off-odor categories (DS + DF). Reserve cans were excluded from calibration and preprocessing fitting; the reserve-set comparison was subsequently used for representation assessment and is therefore not presented as independent external validation. Unlike the exploratory PCA in Figure 9, this figure evaluates the supervised classification performance of M, CLR, and LGR under matched validation conditions. In (**a**), the values above the bars are balanced accuracies; blue bars, grouped cross-validation; orange bars, reserve set. In (**b**), cell numbers are counts of cans and the blue shading is proportional to the count.

**Figure 11:**
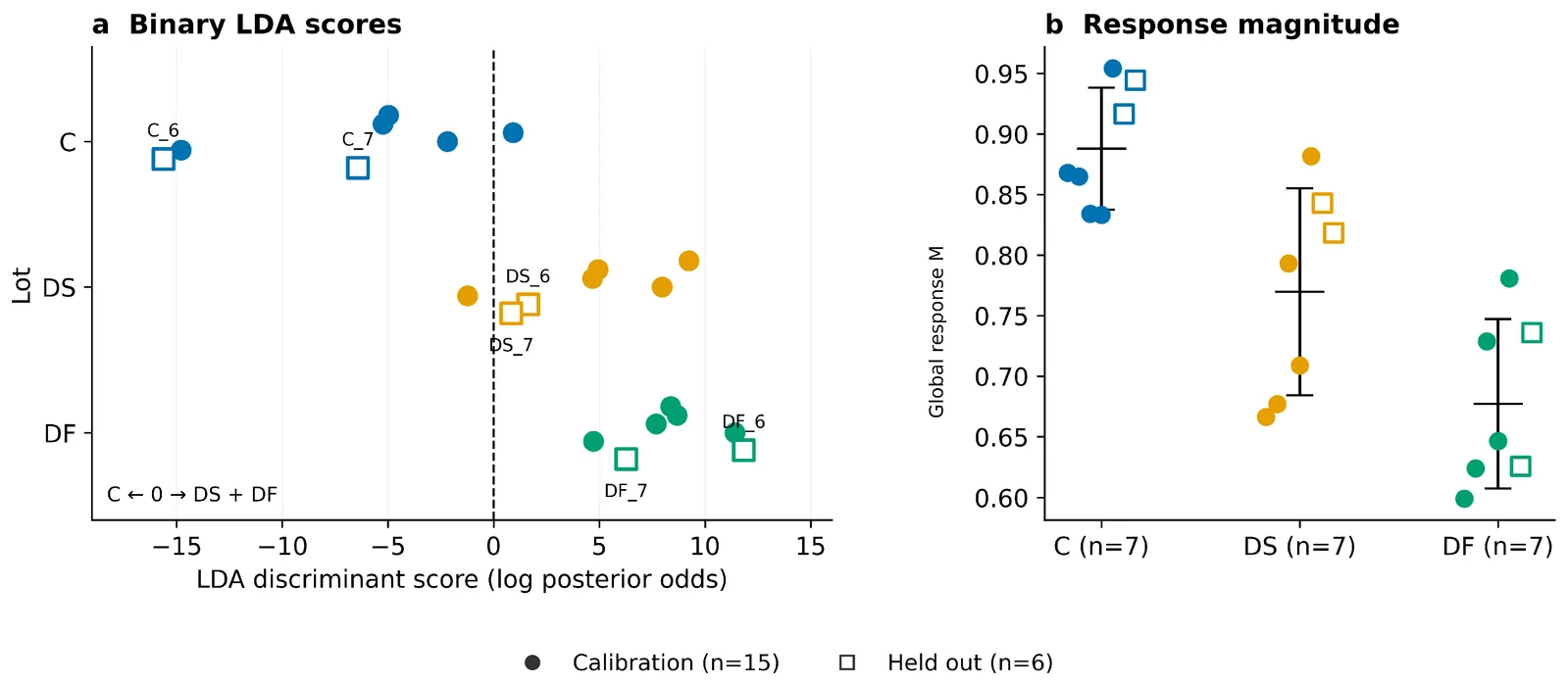
Final LGR-LDA geometry and olive response magnitude. (**a**) Binary LDA discriminant score using the six LGR channels, with scaling and covariance shrinkage fitted on the 15 calibration cans. The score is ln[*P*(D)/*P*(C)]; the dashed line marks zero. The vertical position identifies the supplier category and includes a small display offset only. (**b**) Global magnitude M for the 21 non-coded cans, with individual observations and mean ± standard deviation. Colours identify the supplier categories (blue, C; orange, DS; green, DF). Filled circles: calibration cans; open squares: reserve cans.

**Table 1:** Experimental design and evaluation units.

Dataset	Composition	Sessions	Unit and Purpose
Reference compounds	36 preparations + 6 AGU	6	12 compound × level conditions; 3 preparations per condition
Olive calibration	15 cans: 5 C, 5 DS, 5 DF	4	One row per can; model development
Reserve olives	6 cans: 2 C, 2 DS, 2 DF	S2–S3	Excluded from calibration; within-campaign representation/model comparison
Coded unknowns	3 UNK cans	S4	Coded-prediction demonstration
Olive controls	4 AGU + 8 PAT	4	System verification; excluded from olive class training

Note: C, conforming; DS, mild off-odor; DF, strong off-odor (supplier-assigned categories); AGU, ultrapure-water blank; PAT, ethanol–acetone verification mixture; UNK, coded unknown can; S1–S4, olive measurement sessions.

**Table 2:** Predefined acquisition order for the reference-compound campaign.

Session	Date	Acquisition Order
S1	31 August 2026	AGU → ONA_C1 → ETA_C2 → ACE_C3 → HEX_C1 → ONA_C2 → HEX_C3
S2	2 September 2026	ETA_C1 → ACE_C3 → AGU → HEX_C2 → ACE_C1 → ETA_C2 → ONA_C3
S3	4 September 2026	ACE_C1 → HEX_C3 → ONA_C2 → ETA_C1 → AGU → ETA_C3 → HEX_C2
S4	7 September 2026	HEX_C1 → ONA_C2 → ACE_C2 → ETA_C1 * → ONA_C3 → ACE_C1 → AGU
S5	9 September 2026	ONA_C3 → AGU → HEX_C3 → ONA_C1 → ETA_C2 → ACE_C2 → ETA_C3
S6	11 September 2026	HEX_C2 → ACE_C2 → HEX_C1 → AGU → ETA_C3 → ACE_C3 → ONA_C1

Note: ETA, ethanol; ONA, acetone; ACE, acetic acid; HEX, hexanal; C1, C2, C3, high, intermediate and low nominal concentration levels, respectively; AGU, ultrapure-water blank; S1–S6, reference-compound sessions. * The acquisition initially identified as ETA_C3 on 7 September 2026 was actually prepared as ETA_C1 and was reassigned accordingly. ETA_C3 was repeated with the correct dilution on 9 September 2026. AGU: Milli-Q water. Arrows (→) indicate the chronological order of acquisitions within each session.

**Table 3:** Acquisition order for olives and controls during the four olive sessions.

Session	Date	Acquisition Order	Session Structure
S1	15 September 2026	AGU → PATi → DS_1 → DF_1 → DF_2 → C_2 → DS_2 → PATf	5 calibration cans; C, DS, DF present
S2	16 September 2026	AGU → PATi → DS_3 → DF_6[R] → C_3 → DS_6[R] → DF_3 → C_6[R] → PATf	3 calibration + 3 reserve; one reserve can per category
S3	17 September 2026	AGU → PATi → DF_4 → C_7[R] → DS_4 → DF_7[R] → C_4 → DS_7[R] → PATf	3 calibration + 3 reserve; one reserve can per category
S4	18 September 2026	AGU → PATi → C_5 → UNK_1 → DS_5 → UNK_2 → DF_5 → UNK_3 → PATf → C_1 *	4 calibration + 3 coded unknowns; replacement C_1 measured after PATf

Note: C, conforming; DS, mild off-odor; DF, strong off-odor; AGU, ultrapure-water blank; PATi/PATf, ethanol–acetone verification mixture at session start/end; [R], reserve can excluded from calibration; UNK, coded unknown can; S1–S4, olive measurement sessions. * C_1 is the final replacement calibration can retained in the definitive dataset; it was measured after PATf in S4, and chronological analyses use its actual acquisition date. Arrows (→) indicate the chronological order of acquisitions within each session.

**Table 4:** Nominal concentrations of the reference solutions.

Compound	Family	C1	C2	C3	Unit
Ethanol	Alcohol	0.30	0.10	0.030	% *v*/*v*
Acetone	Ketone	0.100	0.030	0.010	% *v*/*v*
Acetic acid	Carboxylic acid	3.00	1.00	0.30	% *v*/*v*
Hexanal	Aldehyde	50	15	5	mg/L

Note: C1, C2, C3, nominal liquid-phase concentration levels (high, intermediate, low); % *v*/*v*, volume per volume; mg/L, milligrams per liter.

**Table 5:** Signal representations used in the matched comparisons.

Representation	Dimension	Definition	Information Emphasized
M	1	Mean of the six LGR values	Global response magnitude
CLR	6 coordinates (5 independent)	LGR_i_ − M	Relative response profile among sensors
LGR	6	ln(G_i_/G_0,*i*_)	Magnitude + relative profile

Note: LGR_i_, log-conductance ratio of channel i, ln(G_i_/G_0,*i*_), where G_i_ and G_0,*i*_ are the conductances at the end of the sample and reference-air phases, respectively; M, global response magnitude (mean of six LGR values); CLR, centered log-ratio profile (LGR_i_ − M).

**Table 6:** Balanced accuracy of the fixed LDA classifier for the reference-solution class.

Representation	Grouped Condition ^1^	Session Held Out ^2^	Level Held Out ^2^	S6 Hold Out ^3^
M	0.500	0.556	0.528	0.625
CLR (6 channels)	0.833	0.889	0.833	0.750
LGR (6 channels)	0.750	0.889	0.778	0.750
R_f_, ΔR, ΔR/R_0_ (18 variables)	0.750	0.917	0.778	0.750

Note: BA, balanced accuracy; LDA, linear discriminant analysis; M, global response magnitude; CLR, centered log-ratio profile; LGR, log-conductance ratio; Rf, resistance at the end of sample exposure; R_0_, reference-air baseline resistance; ΔR, R_0_ − Rf; S6, sixth reference-compound session. ^1^ Twelve compound × level decisions after the aggregation of three preparations. ^2^ Thirty-six preparation-level predictions. ^3^ Six preparations; BA is not the same as the proportion correct. Values are exploratory and are not formal tests of model superiority.

**Table 7:** Matched LDA comparison of signal representations in olives.

Representation	CV Correct	CV BA	CV MCC	Reserve Correct	Reserve BA	Reserve MCC
M	13/15	0.900	0.756	4/6	0.750	0.500
CLR	13/15	0.850	0.700	5/6	0.875	0.707
LGR	13/15	0.850	0.700	6/6	1.000	1.000

Note: CV, grouped five-fold cross-validation by can; Reserve, six cans excluded from calibration; BA, balanced accuracy; MCC, Matthews correlation coefficient; M, global response magnitude; CLR, centered log-ratio profile; LGR, log-conductance ratio; C, conforming; D, off-odor (DS + DF). In total, 95% Wilson intervals for class sensitivities are broad because of the small numbers of physical cans. Grouped CV: M, C 0.57–1.00 and D 0.49–0.94; CLR/LGR, C 0.38–0.96 and D 0.60–0.98. Reserve set: M, C 0.34–1.00 and D 0.15–0.85; CLR, C 0.34–1.00 and D 0.30–0.95; LGR, C 0.34–1.00 and D 0.51–1.00. These intervals are provided for the class sensitivities rather than for balanced accuracy itself and reinforce that reserve-set ranking is descriptive at *n* = 6.

**Table 8:** Final LGR model evaluation and PLS-DA comparison.

Evaluation	Model	Correct	BA	MCC
Grouped CV by can	LDA	13/15	0.850	0.700
Grouped CV by can	PLS-DA	13/15	0.850	0.700
Reserve set	LDA	6/6	1.000	1.000
Reserve set	PLS-DA	5/6	0.875	0.707
Retrospective chronological	LDA	9/10	0.917	0.816
Retrospective chronological	PLS-DA	8/10	0.833	0.667
Coded unknowns	LDA	3/3 ^1^	—	—
Coded unknowns	PLS-DA	3/3 ^1^	—	—

Note: LDA, linear discriminant analysis; PLS-DA, partial least-squares discriminant analysis; LGR, log-conductance ratio; BA, balanced accuracy; MCC, Matthews correlation coefficient; CV, cross-validation; S1–S4, olive measurement sessions; chronological, model trained on earlier sessions and evaluated on the subsequent session. ^1^ Agreement with the concealed supplier category key. Reserve cans were excluded from calibration; the chronological test aggregates 3, 3, and 4 test cans in the transitions to S2, S3, and S4. The reserve set was used during representation/model assessment and is not an independent external validation set.

**Table 9:** Post hoc sensitivity to an off-odor category omitted from training.

Unseen D Category (Training Categories)	M	CLR	LGR
DF (train C + DS)	7/7	7/7	7/7
DS (train C + DF)	3/7	4/7	4/7

Note: C, conforming; DS, mild off-odor; DF, strong off-odor; D, off-odor (DS + DF); M, global response magnitude; CLR, centered log-ratio profile; LGR, log-conductance ratio; LDA, linear discriminant analysis; sensitivity, proportion of unseen off-odor cans predicted as D.

## Data Availability

The raw MOS electronic-nose acquisitions supporting this study are provided in File_S1_Raw_MOS_Data.zip, together with File_S1_INDEX.csv. The processed M, CLR, and LGR matrices are provided in File_S2_Feature_Matrices.xlsx, and the fixed validation assignments and model predictions are provided in File_S3_Validation_and_Predictions.xlsx. File_S4_README_Data_Dictionary.txt documents the data structure and processing steps, and MANIFEST.md provides file sizes and SHA-256 hashes. NECA ML is an in-house CICYTEX research application created by Ramiro Sánchez Baltasar and is not distributed as source code with this article. The Supplementary Materials provide the software-independent inputs and outputs needed to reproduce or cross-check the statistical analyses in Python, MATLAB/PLS_Toolbox, R, or another suitable chemometric environment.

## Supplementary Materials

The following supporting information can be downloaded at https://www.mdpi.com/article/10.3390/s26206605/s1: File S1: File_S1_Raw_MOS_Data.zip, containing 78 original S3+ acquisitions: 42 reference-solution files arranged in six session folders, 21 olive files in calibration_and_heldout, 12 AGU/PAT control files, and 3 coded-unknown files. The archive includes INDEX.csv (file, dataset, sample code, class, role, and session) and FORMAT.txt, describing the raw-column format and acquisition protocol; File_S1_INDEX.csv is also supplied separately. File S2: File_S2_Feature_Matrices.xlsx, with sheets olives_M_CLR_LGR, patterns_M_CLR_LGR, and variables; each row includes sample identity, session, category, role, usable cycles, chamber-state annotation, M, six CLR variables, six LGR variables, and the six R0 values. File S3: File_S3_Validation_and_Predictions.xlsx, containing a README, five-algorithm comparison tables, out-of-fold predictions with fold assignment for each can, reserve-set predictions for LDA and PLS-DA, chronological-validation predictions, coded-unknown predictions, and the reference-solution prediction tables used in the analysis. Binary machine-readable labels are C and D, where D = DS + DF; manuscript tables report these as Conforming and Defect. File S4: File_S4_README_Data_Dictionary.txt, describing the instrument, sample codes, seven processing steps, the column dictionary for Files S2–S3, and the optional acceptance rule. Figure S1: Figure_S1_NECA_ML_interface (PDF), showing the NECA ML graphical interface; the analytical workflow itself is provided as Figure 4 in the main manuscript. Tables S1–S3: Tables_S1_S3.xlsx, reporting the thermal-regime sensitivity analysis, the post hoc unseen-off-odor-category scope analysis, and the retrospective chronological LDA comparison of M, CLR, and LGR, including transition-level and can-level outputs. MANIFEST.md reports file sizes and SHA-256 hashes for the complete supplementary package. NECA ML itself is not distributed with the Supplementary Materials; the raw acquisitions, exported feature matrices, fixed partitions, model settings, and predictions are supplied so that the reported analyses can be checked independently of the in-house application.
