# Supplementary material for: Disentangling Response Magnitude and Relative MOS Sensor Fingerprints for Electronic-Nose Screening of Off-Odor in Table Olives
Source: Sensors (Basel). 2026 Oct 18;26(20):6605. doi: 10.3390/s26206605 (PMC13709223; doi:10.3390/s26206605)
Supplement: Supplementary file 1 [file sensors-26-06605-s001.zip › Figure_S1_NECA_ML_interface.pdf]

Clasificación · model comparison, grouped CV, confusion matrix of the selected model

NECA ML B0.9.19 · nariz electrónica

NECA ML
Ramiro Sánchez Baltasar
calidad de alimentos · aprendizaje automático
CICYTEX

1 Proyecto
2 Configuración del ensa
3 Muestras y metadatos
4 Señales y extracción
5 Clasificación
6 Validación y desconoci
7 Informes y asistente
8 PLS cuantitativo (opcio
9 Control y deriva
10 Correcciones y diseño
11 Biblioteca NECA
12 Ayuda y glosario

B0.9.19 · compilación 0.9.19b
Ramiro Sánchez Baltasar · CI(

Clasificación

15 determinaciones entrenan (36 en total, otros roles: 8 patron_referencia, 6 validacion_externa, 4 blanco, 3 desconocida). Sólo CALIBRACION entra en el PCA, el DI y el entrenamiento; la réplica con ⚠

☐ Compensar temperatura y humedad de cámara (se estima dentro de cada fold)

Adóptela solo si la validación agrupada mejora; no es un ajuste cosmético.

Unidad que no puede repartirse entre folds: sample_id · muestra física

☒ Modo binario Grupo 0 · nombre: CONFORME clases: C Grupo 1 · nombre: DEFECTO clases: DF, DS

Ver decisión binaria | Ver ROC CV | Separe clases con comas; las no indicadas quedan fuera de este análisis.

Variables: 6 · log(G/G0) · Sensores: los seis · Muestras: 15 entrenan · 6 validación externa · 3 desconocidas · 8 patrón · 4 blanco (36 determinaciones) · Validación: CV agrupada por muestra física, 5 particiones · Modo: binario

Comparación | Matriz de confusión CV | Métricas por clase | Predicciones CV | Ficha técnica | Historial (1)

Modelo	Estado	Peor Ba (Cv/Sesión	Ba Cv	Mcc	F1 Macro Cv	Accuracy Calibración	Error
LDA	PRELIMINAR_PENDIENTE_VALIDACION_EXTI	0.850	0.850	0.700	0.850	0.867	
kNN	PRELIMINAR_PENDIENTE_VALIDACION_EXTI	0.850	0.850	0.700	0.850	1.000	
ANN	PRELIMINAR_PENDIENTE_VALIDACION_EXTI	0.800	0.950	0.577	0.928	0.933	
SVM	PRELIMINAR_PENDIENTE_VALIDACION_EXTI	0.800	0.900	0.577	0.861	0.933	
PLS-DA	PRELIMINAR_PENDIENTE_VALIDACION_EXTI	0.700	0.850	0.400	0.850	0.933	

Matriz de confusión CV del modelo seleccionado · filas: clase real, columnas: predicha · unidad: muestra física

Index	Pred Conforme	Pred Defecto
Real_CONFORME	4	1
Real_DEFECTO	1	9

Scores of the selected model (LDA, two classes: LD1 × first principal component of the residual)

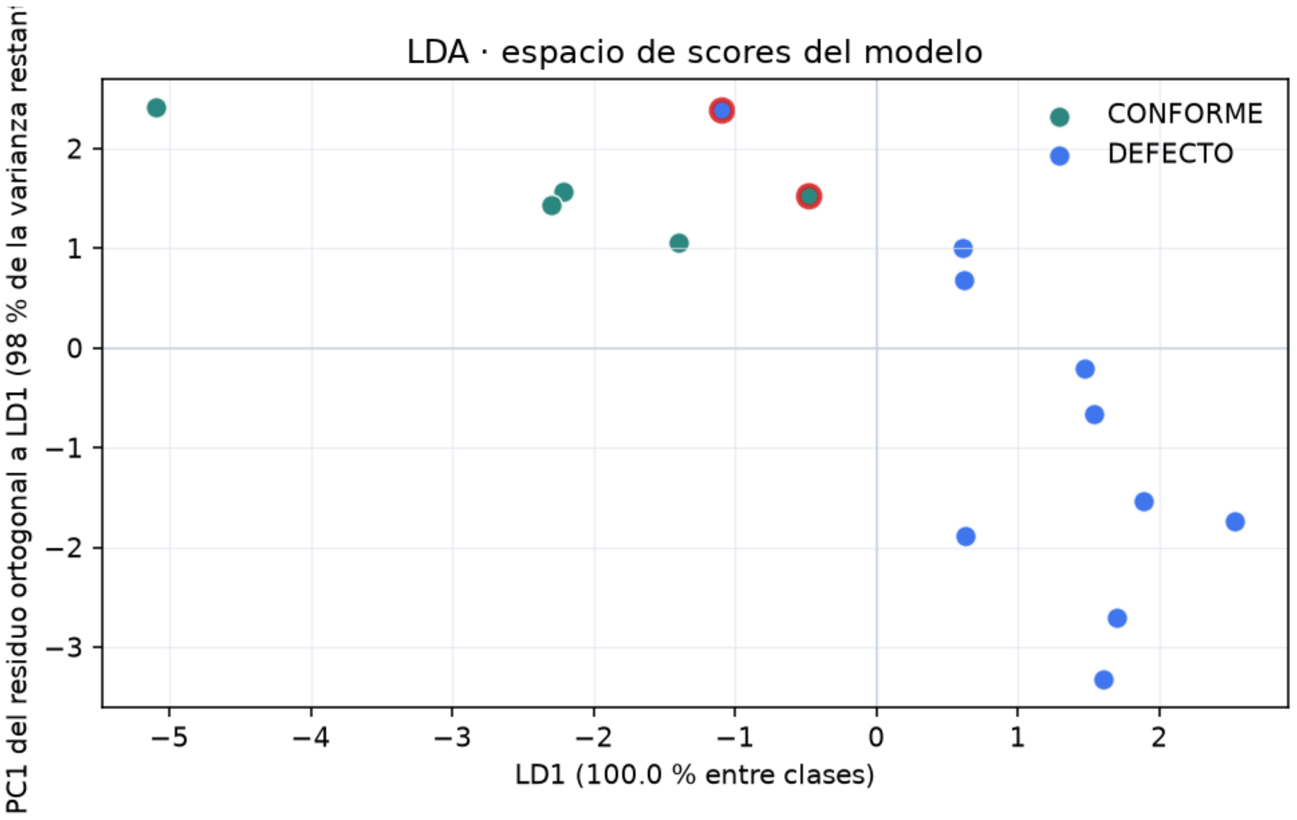

Espacio discriminante LDA descriptivo; la capacidad predictiva se juzga con la validación agrupada. Con dos clases sólo hay un eje discriminante; el eje vertical es la componente principal de lo que LD1 no usa. Una marca por muestra física (n = 15, media de 15 determinaciones). Borde rojo: error en CV agrupada (2 de 15). Ejes ajustados con todas las muestras: la separación dibujada es optimista por construcción. DI geométrico NECA (2σ) = -4.7 sobre los ejes del modelo (optimista: ejes ajustados con todas las muestras); no equivalencia propietaria certificada.

Validación · held-out samples: forced class, confidence and acceptance

NECA ML B0.9.19 · nariz electrónica

NECA ML
Ramiro Sánchez Baltasar
calidad de alimentos · aprendizaje automático
CICYTEX

1 Proyecto
2 Configuración del ensa
3 Muestras y metadatos
4 Señales y extracción
5 Clasificación
6 Validación y desconoci
7 Informes y asistente
8 PLS cuantitativo (opcio
9 Control y deriva
10 Correcciones y diseño
11 Biblioteca NECA
12 Ayuda y glosario

B0.9.19 · compilación 0.9.19b
Ramiro Sánchez Baltasar · CI(

Validación y desconocidas

Validar carpeta externa | Exportar validación | Guardar modelo con validación

☐ Cada CSV externo es una muestra física independiente (marcar solo si no son réplicas de la misma preparación)

La carpeta debe conservar las clases reales en los nombres. Se informa rendimiento sobre todas las muestras, cobertura y rendimiento de las aceptadas por calidad/dominio, manteniendo siempre el denominador completo.

2B · Clasificación de muestras desconocidas

Clasificar desconocidas | Exportar desconocidas | Ver gráfico | Estado operativo | NECA Live (pantalla de planta)

Aquí la clase no se usa porque es desconocida. Se informa la clase propuesta, confianza, dominio, distancia de Mahalanobis y similitud del fingerprint; una muestra fuera de dominio no debe convertirse en decisión automática.

Exportar matrices | Salida técnica opcional de las carpetas ya procesadas; no valida por sí sola.

Validación externa | Predicciones externas | Matriz de confusión externa | Desconocidas por muestra | Desconocidas por archivo | Desconocidas por clase

Muestra Fisica	Clase Real	Clase Predicha	N Replicas	Metodo Agregacic	Confianza Media	Predicciones Replicas	Prediccion Aceptac	Criterio Aceptacior
C_6	CONFORME	CONFORME	1	voto_mayoritario	1.000	CONFORME	Sí	todas_las_replicas_a
C_7	CONFORME	CONFORME	1	voto_mayoritario	0.998	CONFORME	Sí	todas_las_replicas_a
DF_6	DEFECTO	DEFECTO	1	voto_mayoritario	1.000	DEFECTO	No	todas_las_replicas_a
DF_7	DEFECTO	DEFECTO	1	voto_mayoritario	0.998	DEFECTO	No	todas_las_replicas_a
DS_6	DEFECTO	DEFECTO	1	voto_mayoritario	0.835	DEFECTO	No	todas_las_replicas_a
DS_7	DEFECTO	DEFECTO	1	voto_mayoritario	0.696	DEFECTO	No	todas_las_replicas_a

Validación · unknown samples: forced class and inconclusive status

NECA ML B0.9.19 · nariz electrónica

NECA ML
Ramiro Sánchez Baltasar
calidad de alimentos · aprendizaje automático
CICYTEX

1 Proyecto
2 Configuración del ensa
3 Muestras y metadatos
4 Señales y extracción
5 Clasificación
6 Validación y desconoci
7 Informes y asistente
8 PLS cuantitativo (opcio
9 Control y deriva
10 Correcciones y diseño
11 Biblioteca NECA
12 Ayuda y glosario

B0.9.19 · compilación 0.9.19b
Ramiro Sánchez Baltasar · CI(

Validación y desconocidas

Validar carpeta externa | Exportar validación | Guardar modelo con validación

☐ Cada CSV externo es una muestra física independiente (marcar solo si no son réplicas de la misma preparación)

La carpeta debe conservar las clases reales en los nombres. Se informa rendimiento sobre todas las muestras, cobertura y rendimiento de las aceptadas por calidad/dominio, manteniendo siempre el denominador completo.

2B · Clasificación de muestras desconocidas

Clasificar desconocidas | Exportar desconocidas | Ver gráfico | Estado operativo | NECA Live (pantalla de planta)

Aquí la clase no se usa porque es desconocida. Se informa la clase propuesta, confianza, dominio, distancia de Mahalanobis y similitud del fingerprint; una muestra fuera de dominio no debe convertirse en decisión automática.

Exportar matrices | Salida técnica opcional de las carpetas ya procesadas; no valida por sí sola.

Validación externa | Predicciones externas | Matriz de confusión externa | Desconocidas por muestra | Desconocidas por archivo | Desconocidas por clase

Codigo Muestra	N Replicas	Clase Forzada Vot	Concordancia Repl	Concordancia Disp	Resultado Muestra	Predicciones Replicas	Resultados Replica	Deriva Mediana Z	Estado Dominio	Similituc
UNK_1_r1	1	DEFECTO	—	No	NO_CONCLUYENTE_POR_UMBRALES	DEFECTO	NO CONCLUYENTE	1.339	DENTRO_DEL_DOMI	71
UNK_2_r1	1	DEFECTO	—	No	NO_CONCLUYENTE_POR_UMBRALES	DEFECTO	NO CONCLUYENTE	0.699	DENTRO_DEL_DOMI	48
UNK_3_r1	1	CONFORME	—	No	CONFORME	CONFORME	CONFORME	0.274	DENTRO_DEL_DOMI	82
